# A Factor Linking Floral Organ Identity and Growth Revealed by Characterization of the Tomato Mutant *unfinished flower development* (*ufd*)

**DOI:** 10.3389/fpls.2016.01648

**Published:** 2016-11-07

**Authors:** Sandra Poyatos-Pertíñez, Muriel Quinet, Ana Ortíz-Atienza, Fernando J. Yuste-Lisbona, Clara Pons, Estela Giménez, Trinidad Angosto, Antonio Granell, Juan Capel, Rafael Lozano

**Affiliations:** ^1^Centro de Investigación en Biotecnología Agroalimentaria, Universidad de AlmeríaAlmería, Spain; ^2^Laboratorio de Genómica de Plantas y Biotecnología, Instituto de Biología Molecular y Celular de Plantas, Consejo Superior de Investigaciones Científicas, Universidad Politécnica de ValenciaValencia, Spain

**Keywords:** flower development, organ growth, phytohormones, transcriptome, *Solanum lycopersicum* L., *UFD* gene

## Abstract

Floral organogenesis requires coordinated interactions between genes specifying floral organ identity and those regulating growth and size of developing floral organs. With the aim to isolate regulatory genes linking both developmental processes (i.e., floral organ identity and growth) in the tomato model species, a novel mutant altered in the formation of floral organs was further characterized. Under normal growth conditions, floral organ primordia of mutant plants were correctly initiated, however, they were unable to complete their development impeding the formation of mature and fertile flowers. Thus, the growth of floral buds was blocked at an early stage of development; therefore, we named this mutant as *unfinished flower development* (*ufd*). Genetic analysis performed in a segregating population of 543 plants showed that the abnormal phenotype was controlled by a single recessive mutation. Global gene expression analysis confirmed that several MADS-box genes regulating floral identity as well as other genes participating in cell division and different hormonal pathways were affected in their expression patterns in *ufd* mutant plants. Moreover, *ufd* mutant inflorescences showed higher hormone contents, particularly ethylene precursor 1-aminocyclopropane-1-carboxylic acid (ACC) and strigol compared to wild type. Such results indicate that *UFD* may have a key function as positive regulator of the development of floral primordia once they have been initiated in the four floral whorls. This function should be performed by affecting the expression of floral organ identity and growth genes, together with hormonal signaling pathways.

## Introduction

Besides its importance as a major vegetable species, tomato (*Solanum lycopersicum* L.) has been considered as a model species for studying the genetic and hormonal regulation of reproductive development. However, most studies on this topic have focused on the development and ripening of tomato fruit (Lozano et al., 2009; Pesaresi et al., 2014; Azzi et al., 2015) and less attention has been given to the study of floral organogenesis compared to that given to other model plant species such as Arabidopsis, Antirrhinum, and rice. In Arabidopsis, once the floral transition takes place, the shoot apical meristem (SAM) acquires an inflorescence meristem (IM) fate allowing the formation of floral meristems (FM) on its flanks. Genetic control of FM initiation includes, as a main measure, the acquisition of the floral identity by the activation of the FM identity genes *LEAFY* (*LFY*) and *APETALA1* (reviewed in Siriwardana and Lamb, 2012; Winter et al., 2015). Tomato plants exhibit a sympodial growth pattern where the SAM converts into an IM, and vegetative development continues from a specialized axillary meristem called sympodial meristem, which arises from the axil of the last leaf. Meanwhile, the IM produces a new IM on its side before differentiating into a FM. Reiteration of IMs that are arranged perpendicular to one another, results in the production of an inflorescence organized in a zig-zag pattern (reviewed in Lozano et al., 2009; Thouet et al., 2012). *FALSIFLORA* has been characterized as the *LFY* ortholog in tomato where it plays similar functions as *LFY* as a regulator of FM identity (Molinero-Rosales et al., 1999; Thouet et al., 2012). However, *MACROCALYX (MC)*, the tomato *AP1* homolog, most likely regulates IM instead of FM fate in tomato (Vrebalov et al., 2002; Yuste-Lisbona et al., 2016).

Once FM has been determined, floral organ identity is established from specific meristematic domains, which result in the initiation of sepal, petal, stamen, and carpel primordia in the four whorls forming the flowers of most angiosperms. This process is regulated by several overlapping gene functions which act in accordance with the ABC(DE) model (Coen and Meyerowitz, 1991; Causier et al., 2010). Most of the ABC identity functions are carried out by members of the homeotic MADS-box gene family. They encode transcriptional factors, which form part of multimeric protein complexes capable of binding to the target genes responsible for the morphological features of mature floral organs (Honma and Goto, 2001; Smaczniak et al., 2012). In tomato, phenotypic characterization and expression pattern analyses performed in floral homeotic mutants and transgenic plants with affected MADS-box gene expression seem to confirm the ABC(DE) model. Thus, the *MC* A-class gene is, amongst others, involved in sepal development (Vrebalov et al., 2002; Yuste-Lisbona et al., 2016). B-class gene function is carried out by two paralogous of *APETALA3*, i.e., *Tomato AP3* [*TAP3*; syn. *STAMENLESS* (*SL*)] and *Tomato MADS box 6* (*TM6*), and two *PISTILLATA* (*PI*) homologs, i.e., *Tomato PISTILLATA* (*TPI*; syn. *SlGLO2*) and *S. lycopersicum GLOBOSA* (*SlGLO*; syn. *SlGLO1, LePI, TPIB*; Pnueli et al., 1991; Kramer et al., 1998; Busi et al., 2003; de Martino et al., 2006; Leseberg et al., 2008; Geuten and Irish, 2010; Quinet et al., 2014). The *TOMATO AGAMOUS 1* (*TAG1*) gene specifies stamen and carpel identity (Pnueli et al., 1994a) and together with *TOMATO AGAMOUS LIKE-1* (*TAGL1*; syn. *ARLEQUIN* (*ALQ*) (Vrebalov et al., 2009; Giménez et al., 2010), represent C-class gene function, while *TM5* (Pnueli et al., 1994b) and *TM29* (syn. TAGL2; Ampomah-Dwamena et al., 2002; Busi et al., 2003) have been proposed as E-class genes. The *TAGL11* gene represents D class gene function in tomato and is involved in flower and mainly in fruit development (Busi et al., 2003).

Floral organ development includes not only the meristematic signals of the ABC(DE) network which indicate the correct place where a tissue or organ will differentiate from the FM, but also changes in cell proliferation leading to the growth of floral organ primordia. This program initiates in response to meristematic signals and interacts with the organ identity genes (Reinhardt, 2005; Dornelas et al., 2011; Wellmer et al., 2014). As a result, each organ primordia grows initially by cell division and, subsequently, by cell expansion until it acquires its final size and shape (Anastasiou and Lenhard, 2007; Bögre et al., 2008). Members from the ovate family protein (OFP) have been characterized as regulators of cell proliferation. Thus, OVATE-like proteins act as transcriptional repressors in Arabidopsis, mainly affecting the expression of *GIBBERELIN 20 OXIDASE 1*, a key player in the gibberellin biosynthesis, causing a reduction in cell elongation (Hackbusch et al., 2005; Wang et al., 2007, 2011). In tomato, the *OVATE* gene acts as a negative regulator of growth, since the loss-of-function *ovate* mutant exhibits elongated carpels (Liu et al., 2002). Likewise, significant changes in cell division are due to a mutation in the tomato cell cycle-control gene *FRUIT WEIGHT 2.2* (*FW2.2*), which encodes a negative regulator of this process (Frary et al., 2000). On the contrary, *SUN* is a member of the IQD family of calmodulin-binding proteins and acts as a positive regulator of growth in tomato, since its high expression level is associated with increases in cell division (Xiao et al., 2008, 2009).

Phytohormones participate in the initiation and outgrowth of floral organ primordia such as auxins that play a key role in the arrangement of floral organs (Wellmer et al., 2014). The unraveling of the genetic network and molecular mechanisms which control the dynamics of flower development, and particularly, the link between identity and growth of floral organs, is an important goal of plant biology currently (Jaeger et al., 2013) and some of the players involved in this process have begun to be identified in Arabidopsis (reviewed in Dornelas et al., 2011; Ó'Maoiléidigh et al., 2014; Wellmer et al., 2014). Extensive genetic and molecular analyses have shown that the homeotic MADS box proteins involved in the ABC model play a master role during both organ identity determination and organ morphogenesis (reviewed in Dornelas et al., 2011; Wellmer et al., 2014). Recently, genome-wide approaches have led to substantial progresses in the identification of genes functioning downstream in the signaling pathway triggered by MADS box proteins (Wellmer et al., 2004, 2006; Kaufmann et al., 2009, 2010; Yant et al., 2010; Wuest et al., 2012; Ó'Maoiléidigh et al., 2013; Pajoro et al., 2014). Many of these genes code for transcription factors or are involved in hormone biosynthesis and signaling (Dornelas et al., 2011; Wellmer et al., 2014). Thus, the floral organ identity factors appear to mediate floral organogenesis by controlling the expression of other regulatory genes, whereas it is though that different phytohormones have a key function in the initiation and differentiation of floral organs. Most phytohormones were indeed shown to be involved in floral organ development in Arabidopsis (Chandler, 2011; Yuan and Zhang, 2015; Marsch-Martínez and de Folter, 2016). Auxin, gibberellin, and jasmonic acid are involved in petal development through the regulation of the *BIGPETAL* transcription factor (Chandler, 2011). During stamen development, jasmonic acid interacts with auxins and gibberellins to regulate anther development and pollen maturation (Plackett et al., 2011; Yuan and Zhang, 2015). Gibberellins also control filament elongation (Plackett et al., 2011). In addition to these major regulators of stamen development, ethylene has been shown to be involved in stamen initiation and cytokinins and brassinosteroids are required for proper pollen development (Chandler, 2011). Regarding gynoecium development, auxins promote carpel initiation, gynoecium growth, and proper style and stigma formation while cytokinins participate in the development of the carpel margin meristem and derived tissues as well as in the valve margin formation (Marsch-Martínez and de Folter, 2016). Finally, after fertilization, synthesis, and signaling of gibberellins are induced to trigger fruit growth (Gallego-Giraldo et al., 2014). However, although progress has been made in the recent years in the understanding of the hormonal control of floral organ development, we are not yet at the stage of having precise networks between hormone signaling pathways and floral organ identity and building gene interactions for the different floral organ developmental stages.

The genetic mechanisms underlying floral organ growth remain largely unknown, particularly in tomato, where genes specifically involved in this developmental process have not been discovered hitherto. The tomato *unfinished flower development* (*ufd*) mutant has been identified from an EMS-mutagenized M2 population of tomato (cv. Moneymaker). The *ufd* flowers are unable to complete the normal growth of floral organ primordia, even though they were correctly initiated in their corresponding floral whorls. We recently characterized the genetic interactions of *ufd* with other tomato mutants showing defects in diverse processes related to inflorescence and flower development, which showed that *UFD* might play a pivotal role between inflorescence architecture and flower initiation genes (Poyatos-Pertíñez et al., 2016). In this paper, we further characterized *ufd* mutant to gain insight in the role of *UFD* during reproductive development of tomato. Transcriptome analysis indicated that *UFD* has a key role in the genetic control of floral organogenesis by affecting the expression of floral organ identity and cell cycle genes, and plant hormones. To provide additional insights into the regulatory interactions of *UFD* with floral organ identity and growth genes, expression analyses were carried out in *ufd* and wild type (WT) flowers during inflorescence development. Moreover, the complete hormonal profile was investigated in *ufd* flowering shoot apices to highlight the involvement of *UFD* in hormonal metabolism and the role of hormones in tomato flower development. All together our results indicated that *UFD* has a key function as positive regulator of floral organ identity and growth, presumably by regulating transcriptional activity and hormonal signaling pathways.

## Materials and methods

### Plant material and growth conditions

The *ufd* mutant was isolated from an M2 population of tomato (*S. lycopersicum* L., cv. Moneymaker), which was generated by mutagenesis with ethyl methanesulphonate (EMS). Genetic analysis of *ufd* mutation was initially performed on 20 plants of the original M2 family, and subsequently in a population composed of 543 M3 plants obtained by selfing a single heterozygous M2 plant (genotype+/*ufd*). Phenotype segregations were verified by a χ^2^-test.

Seeds were germinated in plastic pots filled with sphagnum, peat/coco, peat/vermiculite substrate mixture (3:2:1). Plants were grown under standard greenhouse conditions, 25°C daytime maximum to 10°C night minimum temperature under ~14 h natural light. All plants received regular watering and fertilizer treatments.

### Microscope analysis

Fresh plant tissues (inflorescences, flowers, floral buds, and floral organs) were dissected and examined with a Nikon SMZ445 stereomicroscope to determine morphological and developmental characteristics. For histological studies, inflorescence samples were harvested and treated in FAA (3.7% formaldehyde, 5% acetic acid, 50% ethanol) for 2 h under partial vacuum conditions and then kept for 20–24 h at room conditions. For light microscopy, fixed samples were dehydrated in an ethanol series (70–100%) and embedded in paraffin (Paraplast plus, Sigma-Aldrich). Sections (8 μm-thick) were cut with a Leica RM2145 microtome and stained using eosin. Later, paraffin was removed and tissue sections were dehydrated in an ethanol series, stained in a 1% Blue Toluidine solution and analyzed and photographed with a Nikon Optiphot2 microscope. Scanning electron microscopy (SEM) analysis was carried out as follows: samples were fixed in FAEG (3.7% formaldehyde, 5% acetic acid, 50% ethanol, and 0.5% glutaraldehyde) and kept in 70% ethanol. Samples were dehydrated in an ethanol with increasing concentrations (70–100%) and them critical-point dried in liquid CO_2_. Subsequently, samples were gold coated and observed using a Hitachi S-3500N scanning electronic microscopy machine at an accelerating voltage of 10 kv.

### Transcriptome analysis by microarray hybridization

In order to examine the differential gene expression patterns in wild type and *ufd* inflorescences, RNA samples were obtained from pooled flowering shoot apices of the last initiated inflorescence harvested on 40-day-old plants of each wild type and *ufd* genotypes, in which the oldest flower buds were around stage 4 (according to Brukhin et al., 2003). Three independent biological replicates per genotype were performed, each one with at least 20 inflorescence apices (4–5 mm long). The six RNA samples were reverse transcribed and labeled with Cy5. Equal aliquots of RNA from the six different samples were mixed and labeled with Cy3 to create a common reference RNA sample. An aliquot of the common reference sample was mixed with each labeled test cDNA sample and the subsequent probes were then hybridized to TOM2 oligo-arrays as previously described (Lytovchenko et al., 2011). The same reference sample was used to hybridize each slide, which enabled the direct comparison of all hybridization experiments. Raw data files are available with accession number E-MTAB-5144 at ArrayExpress (www.ebi.ac.uk/arrayexpress/). The mean LOWESS-normalized values for a gene between wild type and *ufd* samples was compared by one-way ANOVA in order to identify differentially expressed genes. An adjusted *P*-value cut-off of 0.05 was used to identify genes as differentially expressed between experimental groups. A hierarchical clustering was constructed using Pearson correlation coefficient as a similarity metric and the logarithm ratios of the fold change values for each gene in each experimental group as the input data.

### Quantitative real-time PCR analysis

Gene expression analyses were performed by quantitative real-time PCR (qRT-PCR) experiments on wild type and mutant inflorescences at three stages of development: (i) flowering shoot apex containing the last initiated inflorescence, where the oldest flower buds was around stage 4 (according to Brukhin et al., 2003); (ii) type 1 inflorescence (IF1) corresponded to the penultimate initiated inflorescence, including flower buds from stages 6 to 12 in WT and up to stage 5 in *ufd*, since the growth of floral buds is arrested at this stage; and (iii) type 2 inflorescence (IF2) that is the third inflorescence from the top, which contained floral buds from stages 8–18 in WT while in the *ufd* IF2 only included flower buds up to stage 5. Total RNA was isolated using the Trizol Kit (Invitrogen) according to the manufacturer's recommendations. Contaminating DNA was removed using the DNA-free™ kit (Ambion). Subsequently, M-MLV Reverse transcriptase kit (Invitrogen) was used to synthesize first strand cDNA. The reaction was performed according to the recommended protocol by using an oligo (dT) primer and 1 μg RNA. Specific primers for each examined gene are described in Supplementary Table 1. SYBR Green PCR Master Mix kit (Applied Biosystems) was used to perform qRT-PCR reactions in a 7300 Real-Time PCR System (Applied Biosystems), according to the manufacturer's instructions. For each inflorescence stage, qRT-PCRs with three biological and two technical replicates were performed. Seven thousand and three hundred System Sequence Detection Software v1.2 (Applied Biosystem) was used for data collection and analysis of expression levels. Results were processed using the ΔΔCt calculation method, expressed in arbitrary units and normalized by comparison to the housekeeping *UBIQUITINE3* (*UBI3*) gene and then to the value of the WT control. A Student's *t*-test was used for the comparison of data sample means and probability values <0.05 were considered statistically significant (*P* < 0.05).

### Hormonal analysis

Concentrations of the endogenous phytohormones including cytokinins, auxins, gibberellins, abscisic acid, salicylic acid, jasmonic acid, ethylene precursor 1-aminocyclopropane-1-carboxylic acid (ACC), brassinosteroids, benzoic acid, and their metabolites were determined in developing inflorescences (pooled flowering shoot apices harvested on 40-day-old plants) of *ufd* and WT plants according to Quinet et al. (2014). Phytohormones were extracted using 50–100 mg lyophilised plant material from three biological replicates with methanol/formic acid/water (15:1:4, by volume) and then purified using the dual-mode solid-phase method (Dobrev and Kamínek, 2002). An HPLC Ultimate 3000 (Dionex, Sunnyvale, CA, USA) coupled to a 3200 Q TRAP hybrid triple quadrupole/linear ion trap mass spectrometer (Applied Biosystems, Foster City, CA, USA) was used to perform the analysis and quantification of plant hormone levels by means of a multilevel calibration graph with 2H-labeled internal standards. Shapiro-Wilk and Levene's-test were applied to evaluate the normality and homogeneity of the results, respectively (data were transformed when required). ANOVA and Student's-test were used for statistical analysis to evaluate the significance of the genotype effects on the hormonal composition using SAS 9.2.

## Results

### Phenotype and genetic characterization of *ufd* mutant

**Figure 1:**
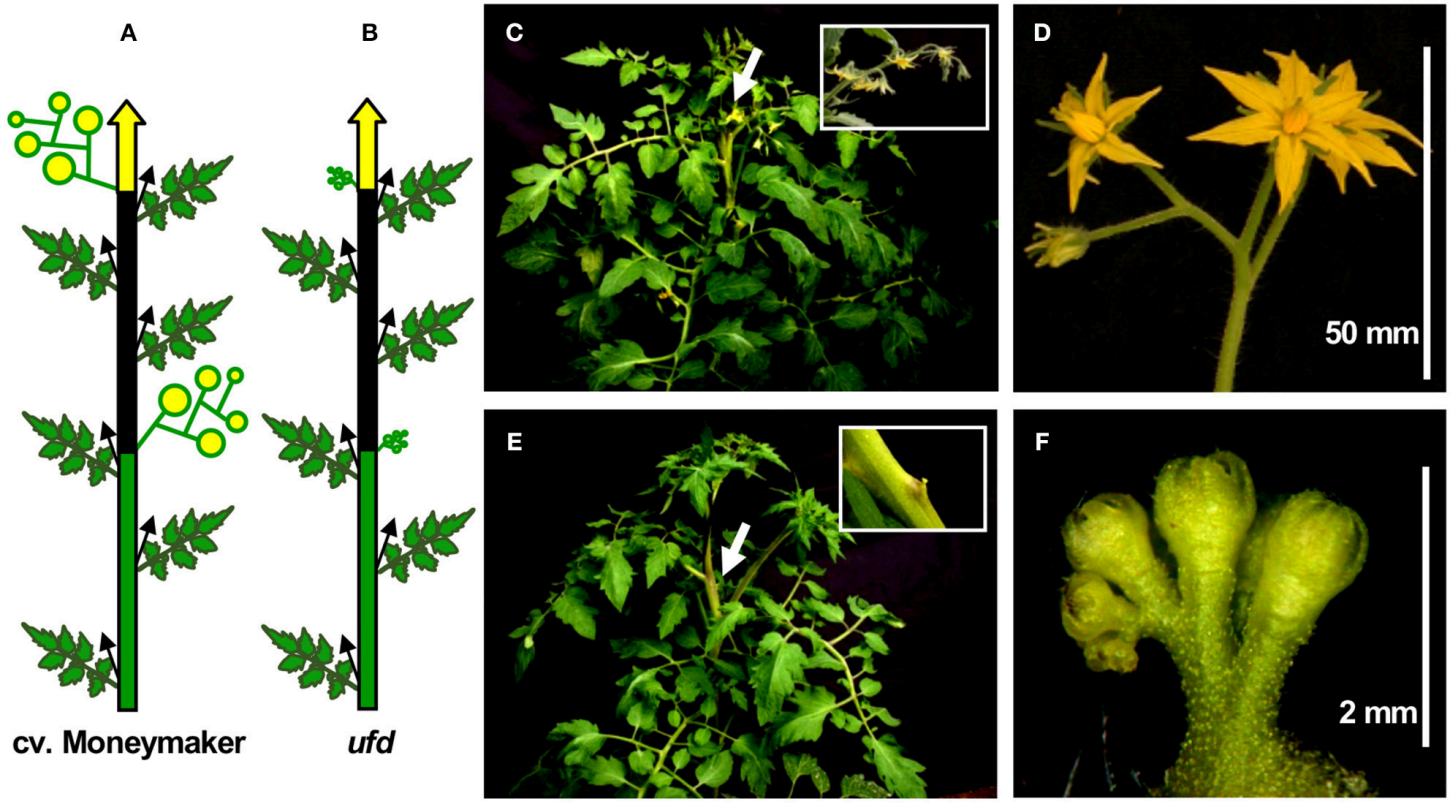
**Structure of wild type and ***unfinished flower development*** (***ufd***) plants grown under standard conditions. (A,B)** Diagrammatic representation of wild type (WT) and mutant tomato plant growth. Green bars represent initial vegetative segment. Sympodial segments are in black. Yellow arrows represent sympodial meristems. Circles symbolize flowers, and black arrows represent developed axillary shoots. **(C,D)** Morphology of a wild type **(C)** and an *ufd* mutant plant **(E)**. White arrows indicate inflorescence position. The insets show a closer view of the inflorescences. **(D,F)** Adult inflorescences of the wild type **(D)** and the *ufd* mutant **(F)**. White bars show inflorescence size.

A screening was performed in an ethyl methanesulfonate (EMS)-induced tomato mutant collection with the aim to isolate new regulatory genes involved in flower development, particularly focused on those mutant phenotypes affected in floral organ identity and growth. As result, a mutant was identified and named *ufd* as both their inflorescences and flowers showed a severe reduction in size and were unable to complete their normal development (Figure 1). As wild type tomato plants (cv. Moneymaker), the primary shoot of *ufd* mutant plants developed an initial vegetative segment composed by 6–7 leaves before the formation of the first inflorescence, and the subsequent plant growth continued through the development of an indeterminate number of sympodial segments, which were normally composed of three leaves and one inflorescence (Figures 1A–C,E). No differences between *ufd* and WT plants were observed in vegetative growth and flowering time (Figure 1 and Supplementary Table 2). After undergoing floral transition, *ufd* plants initiated the development of the inflorescence by the formation of the successive flowers in a zig-zag pattern, as normally occur in wild type tomato plants (Figures 1D,F). Nevertheless, once floral buds were initiated, they were unable to follow a normal growth thus impeding the formation of mature flowers. In addition, the *ufd* plants yielded a smaller number of flowers per inflorescence compared to the WT plants (5.21 ± 0.89 and 7.83 ± 0.89 flowers in *ufd* and WT, respectively, *P* < 0.05).

A genetic analysis was performed in an M2 segregating population, where the *ufd* mutant phenotype was observed in one quarter of plants (15 WT: 5 *ufd*), suggesting a monogenic and recessive inheritance pattern. As the mutation resulted in sterile plants, WT individuals of the same M2 family were selfed, and an M3 segregating population was obtained from a single heterozygous M2 individual. Phenotypic characterization of 543 plants of this M3 progeny revealed a 3:1 ratio for the wild type: mutant phenotype (391: 152, χ^2^ = 2.59, *P* = 0.11), which corroborated that *ufd* phenotype was caused by a monogenic and recessive mutation.

### Floral ontogeny of *ufd* mutant

**Figure 2:**
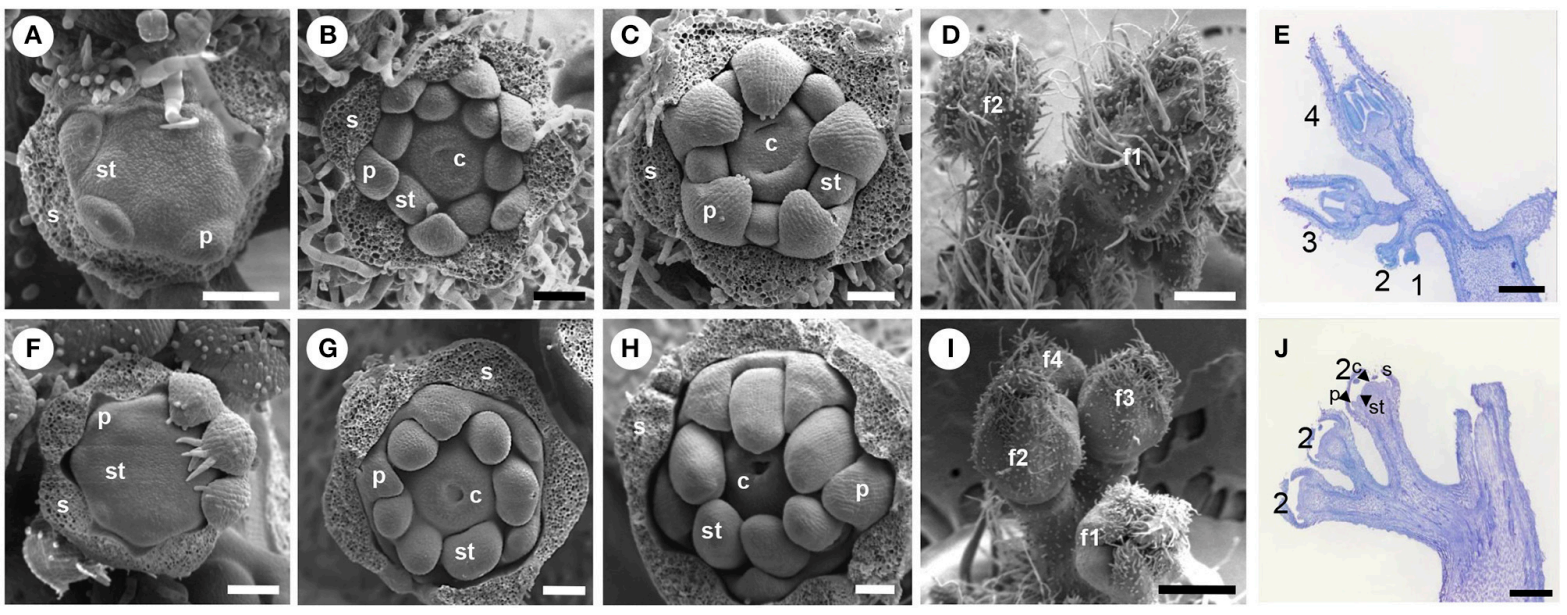
**Scanning electron microscopy (SEM) analysis and histological microscopy of floral development from wild type and ***unfinished flower development (ufd)*** mutant plants. (A–E)** Wild type floral buds. **(F–J)** Mutant flowers. Wild type **(A)** and *ufd* **(F)** floral buds at stage 3, corresponding to stamen primordia initiation (most sepals were removed). Wild type **(B)** and *ufd* **(G)** floral buds at stage 5, when carpel primordia emerge and the distinct ovary cavities are visible (sepals removed). **(C)** Wild type flower at stage 6, when petals bend down to cover reproductive organs and the carpels grow up but are still unfused (sepals removed). **(H)** Floral bud at the most adult stage observed in *ufd* mutant plants (sepals removed). **(D)** Wild type inflorescence with floral buds at stage 6 (f1) and 5 (f2). **(I)** *ufd* inflorescence showing the most developed floral bud at stage 5 (f1) and others at earlier stages (f2–f4). **(E)** Wild type inflorescence with flowers at different development stages (1, 2, 3, and 4). **(J)** *ufd* inflorescence with all flowers showing the same development stage (floral organs stop their development at primordia stage). All developmental stages are classified according to Brukhin et al. (2003). s, sepal; p, petal; st, stamen; c, carpel. Scale bars are 200 μm **(A–C,F–H)**, 500 μm **(D,E,I,J)**.

A detailed analysis of floral organogenesis (Figure 2) was performed on floral buds of *ufd* and WT plants at different developmental stage by SEM, according to those described by Brukhin et al. (2003). At early stages (from stage 3 to 5), *ufd* floral buds were found to develop similarly to those of the wild type background (Figures 2A,B,F,G). As the development progressed, organ primordia were initiated at the expected positions in the corresponding floral whorls. Initially, sepal primordia arose in a helical order. Subsequently, five to six petal primordia appeared simultaneously, followed by stamen and carpel primordia, which alternated with respect to the preceding whorl primordia as described previously (see review of Lozano et al., 2009). Slightly later, and coinciding with the end of stage 5 and stage 6 of tomato flower development (Figures 2C,H), the growth of floral buds is blocked in *ufd* plants (Figures 2I,J) while WT flowers continued to develop (Figures 2D,E), therefore this was considered the “most adult stage” of *ufd* flower development. At this stage, sepal primordia forming the first whorl ceased their development when they were still fused (Figures 2H,I), and trichomes appeared in a lower density on the sepal abaxial surface but restricted to a reduced area around the apical zone (Figures 2D,I). Petal primordia (second whorl) also stopped their development and remained as fused organs as expected for this developmental stage. They did not elongate enough to cover stamen primordia and trichomes were not observed on their abaxial surface (Figure 2H). The third floral whorl in adult *ufd* flowers consisted of dome-shaped stamen primordia, which reached a size comparatively higher than the petals, while carpel primordia led the formation of one or two faint cavities in the innermost whorl (Figure 2H). Moreover, no morphological differences were found in the epidermal cells of mutant floral organs or in their whorled disposition, indicating that *ufd* mutation does not affect morphology or phyllotaxis of floral organs (Figures 2D,E, I,J).

### Transcriptome analysis of *ufd* mutant

To have a wider perspective of the transcriptional changes promoted by the *ufd* mutation during floral development, a global gene expression analysis was performed using the tomato—TOM2 microarray. With this purpose, each of the three biological replicates of the wild type and *ufd* transcriptomes was compared against a common reference sample. After filtering out redundant spots and selecting the ones which were significantly expressed between any of the two samples, a total of 325 transcripts were found to show differential expression in their mRNA levels in *ufd*, corresponding to 4.4% of all spots (7380) which passed quality controls.

The differentially expressed genes (DEGs) identified from the *ufd* vs. WT comparison were assigned to different functional categories (Supplementary Figure 1, Supplementary Tables 3, 4) according to the Tomato Functional Genomics Database (TFGD, http://ted.bti.cornell.edu/). Cellular component categories such as cell, intracellular, and organelle were more represented in the up-regulated gene dataset while the photosystem and thylakoid-light-harvesting complex categories were enriched in the down-regulated gene dataset (Supplementary Figure 1A, Supplementary Tables 3, 4). With regard to their molecular function, the percentage of transcripts classified under categories linked to nucleic acid binding, structural constituent of ribosome, and structural molecule activity were greater in the up-regulated gene dataset (Supplementary Figure 1B, Supplementary Table 3). Categories such as transcription regulator activity, transcription factor activity, and sequence-specific DNA binding were more richly represented in the down-regulated gene dataset (Supplementary Figure 1B, Supplementary Table 4).

**Figure 3:**
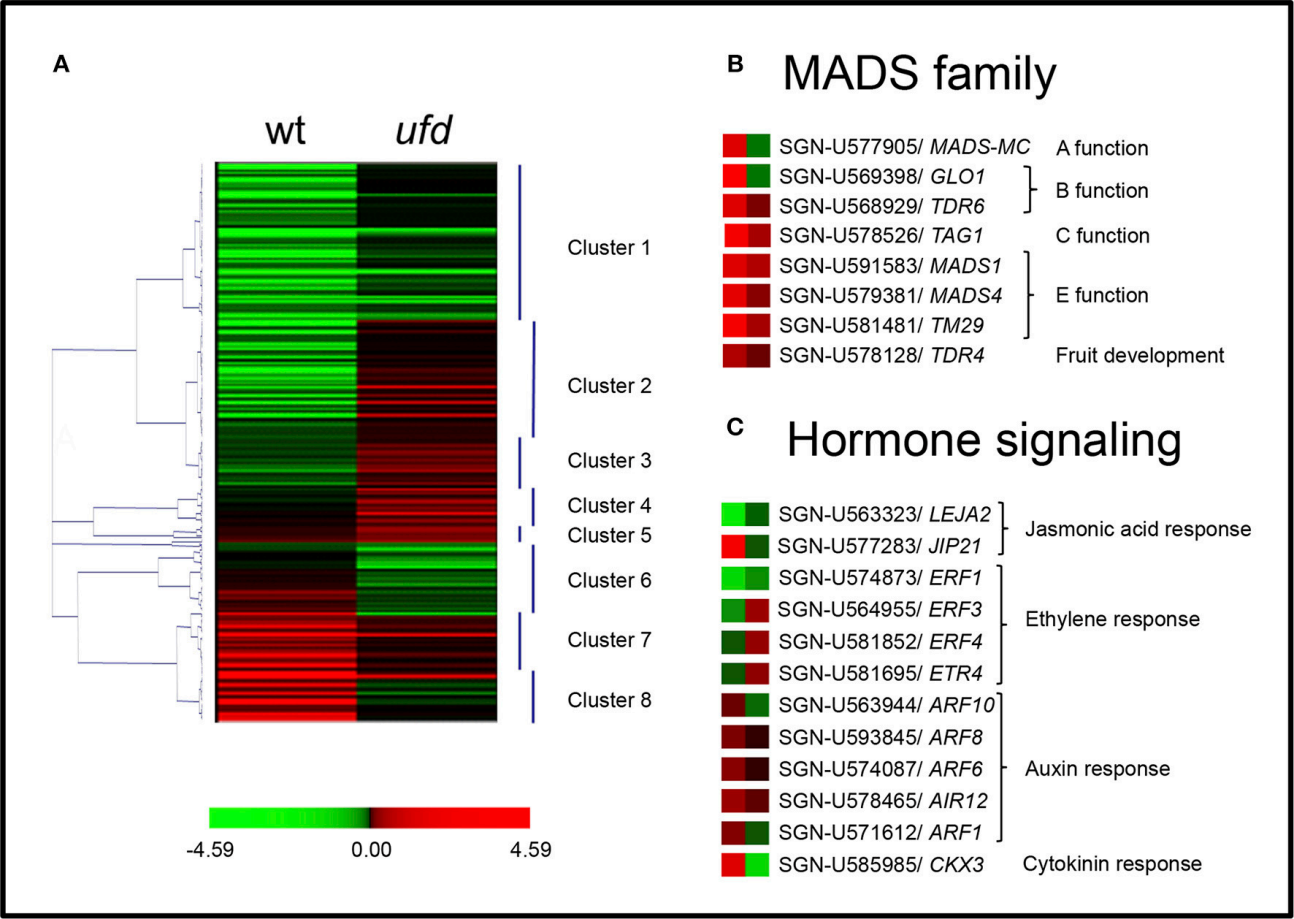
**Expression distribution of genes differentially expressed in ***unfinished flower development (ufd)*** and wild type (WT) tomato flowering shoot apices. (A)** Hierarchical clustering of all genes differentially expressed in the *ufd* mutant compared to WT (see also Supplementary Table 5). **(B,C)** Heat-map of MADS genes and genes involved in hormone signaling, respectively. Color scale indicates logarithm ratio of the fold change in mutant compared with WT. Red color represents genes showing higher expression level and green color indicates reduced expression.

In order to identify genes sharing a similar expression pattern, the whole set of 325 DEGs was clustered into eight clusters (numbered 1–8) according to their differential expression using the Pearson clustering algorithm (Figure 3 and Supplementary Table 5). DEGs in *ufd* samples were grouped into two large clusters (clusters 1 and 2) and several smaller clusters. The largest cluster (cluster 1, 29%) included genes repressed both in wild type and *ufd* mutant samples when compared with the common reference, although at a lower level in the later one. Among others, this cluster included genes encoding transcription factors belonging to WRKY and bHLH families, and others involved in the ethylene pathway such as *ETHYLENE RESPONSE FACTOR 1* and *Lycopersicum esculentum Jasmonic Acid 2* (*LEJA2)*. Cluster 2 (20%) and cluster 4 (6%) included genes showing minor down-regulation in the WT and slight up-regulation in the mutant, although the former showed either greater down-regulation in the WT while the latter showed greater up-regulation in the mutant. Cluster 2 included the elongation factor 1-alpha and some transcription factors, whereas cluster 4 included genes encoding transcription factors of AP2 and WRKY families, together with the *OVATE* gene involved in tomato fruit shape and several ethylene-related genes. Cluster 3 (10%) consisted of mostly constitutive genes showing a similar expression profile to that of cluster 2 and cluster 4. Some representatives of this cluster were genes encoding H2A and H2B histones and protein kinases. Genes included in cluster 7 (14%) showed major up-regulation in wild type inflorescences and minor up-regulation in the mutant ones. Some floral organogenesis-specific genes most of them belonging to the MADS-box family, such as *TDR4, TDR6, LeMADS1, TM29*, and putative orthologs of *CRABS CLAW (CRC)* and *pMADS4* were found in this cluster, together with putative orthologs of *AUXIN RESPONSE FACTOR 8* (*ARF8*), *ARF6*, and the *PHYTOENE SYNTHASE* gene involved in the synthesis of carotenoids. Cluster 5 (5%) were also represented by genes up-regulated both in wild type and *ufd* inflorescences, although differences in the expression level favored the *ufd* inflorescences in this cluster, which was mostly composed by constitutive genes encoding ribosomal proteins.

The loss-of-function phenotype of the *ufd* mutant, which agreed with its recessive inheritance, led us to focus our attention on two additional clusters (Figure 3 and Supplementary Table 5). Cluster 8 (11%) included genes significantly up-regulated in wild type inflorescences but strongly down-regulated in *ufd* mutant ones, among them those encoding MADS-box transcription factors such as *MC* and a *SlGLO1*, as well as other putative orthologs of flowering-related genes as C*YTOKININ OXIDASE 3 (CKX3), ARGONAUTE, ARF1* and an ethylene-responsive transcription factor *RAP2*. Similarly, cluster 6 (5%) contained genes, which were also up-regulated in WT, although at a lower level than in cluster 8, and greatly down-regulated in the *ufd* mutant. Some genes involved in abiotic stresses and in auxin response such as *ARF10* were included in this cluster.

Among the 325 DEGs, a total of 31 and 13 showed more than two-fold up- and two-fold down-regulation in the *ufd* mutant transcriptome, respectively (Table 1). The retrieved set of 31 up-regulated genes appeared not to be significantly enriched in any particular biological or molecular functional category, neither that corresponding to transcriptional regulatory nor developmental processes (Table 1). In this set four transcription factors were encountered, three of them belonging to the WRKY family together with the putative ortholog of the *BRASSINOSTEROID ENHANCED EXPRESSION 3* bHLH transcription factor. Genes also included in this set code for an elongation factor-1 alpha, a calcium-dependent protein kinase, a cytochrome P450, a 2-oxoglutarate-dependent dioxygenase involved in ethylene synthesis, and *LEJA2*. Most of the remaining up-regulated genes were involved in either biotic or abiotic stress responses. The set that was down-regulated in *ufd* inflorescences was particularly enriched in genes encoding for transcription factors of the MADS-box family (Table 1), including *SlGLO1, TM29, MC, TDR6*, and a putative ortholog of *pMADS4*. An additional transcription factor encountered was the putative ortholog of *Nicotiana tabacum CRC*. The rest of the down-regulated genes included *CKX3*, chymotrypsin inhibitor jasmonic-induced protein 21 (JIP1), flower-specific gamma-thionin-like protein/acidic protein precursor and wound-induced proteinase inhibitor 1.

**Table 1:** **Differentially expressed genes showing more than two-fold up- and two-fold down-regulation in ***unfinished flower development*** (***ufd***) relative to wild type plants**.

**Probe ID**	**Gene ID**	**Fold change**	**GenBank ID**	**Annotation**	***e*-value**
LE9L13	SGN-U579680	30.718	P10798	Ribulose bisphosphate carboxylase small chain 3B	1e-32
LE23F03	SGN-U582797	8.938	BAA13150	NT16 polypeptide (*Nicotiana tabacum*)	2e-32
LE15E11	SGN-U571964	8.381	AAF63515	TMV-induced protein I (*Capsicum annuum*)	2e-71
LE26P24	SGN-U580191	8.125	P17786	Elongation factor 1-alpha	0
LE14O13	SGN-U593442	4.746	AAP43673	PR5-like protein (*Lycopersicon esculentum*)	1e-77
LE9D23	SGN-U589805	4.206	CAO39940	Unknown protein	2e-19
LE4M08	SGN-U578841	3.925	Q43502	Proteinase inhibitor type II CEVI57	2e-106
LE6G19	SGN-U580000	3.867	AAG16757	Putative glutathione S-transferase T2 (*Lycopersicon esculentum*)	1e-117
LE13C07	SGN-U576746	3.402	AEC07585	C2H2 zinc finger protein FZF (*Arabidopsis thaliana*)	8e-94
LE7I09	SGN-U578506	3.396	No hits	No hits	
LE3I18	SGN-U581493	3.361	No hits	No hits	
LE29C09	SGN-U563323	3.341	AF011555	LEJA2 Jasmonic acid 2	0
LE31P05	SGN-U570189	3.304	AEC08624	GCN5-related N-acetyltransferase-like protein (*Arabidopsis thaliana*)	3e-47
LE28P15	SGN-U567304	3.295	AAK07676	Non-symbiotic hemoglobin class 1 (*Lycopersicon esculentum*)	2e-080
LE6O08	SGN-U577557	3.269	AAU95238	Osmotin-like protein (*Solanum phureja*)	4e-156
LE25D18	SGN-U577356	3.057	No hits	No hits	
LE26A07	SGN-U579236	2.922	BAD98961	2-oxoglutarate-dependent dioxygenase (*Solanum lycopersicum*)	0
LE17C09	SGN-U579850	2.728	AEE73926	Endoribonuclease Dicer-like 2 (*Arabidopsis thaliana*)	5e-120
LE8C19	SGN-U580303	2.710	NP_849875	MLP-like protein 28 (*Arabidopsis thaliana*)	1e-68
LE17B14	SGN-U571844	2.702	AAA65637	Peroxidase	0
LE26L14	SGN-U581493	2.697	No hits	No hits	
LE31K04	SGN-U580119	2.573	No hits	No hits	
LE31O23	SGN-U583039	2.514	NP_177524	BEE3, bHLH transcription factor (*Arabidopsis thaliana*)	1e-46
LE22P06	SGN-U571844	2.496	AAA65637	Peroxidase (*Solanum lycopersicum*)	0
LE23B10	SGN-U580500	2.428	BAC23031	WRKY-type DNA binding protein (*Solanum tuberosum*)	3e-82
LE33C13	SGN-U576561	2.351	NP_189542	Calcium-binding EF-hand family protein (*Arabidopsis thaliana*)	9e-19
LE6I12	SGN-U571983	2.229	O64882	Beta-glucosidase 17	2e-171
LE17F23	SGN-U577821	2.225	Q05047	Cytochrome P450 (*Catharanthus roseus*)	4e-130
LE18L09	SGN-U580535	2.033	ABM06179	Glutathione transferase, putative	3e-41
LE18E04	SGN-U583014	2.020	BAA89235	WRKY-type DNA binding protein, TMV response-related (*Nicotiana tabacum*)	4e-89
LE3G12	SGN-U596360	2.003	NP_174279	WRKY71; transcription factor (*Arabidopsis thaliana*)	1e-37
LE30P05	SGN-U568620	−2.009	No hits	No hits	
LE20N23	SGN-U568929	−2.058	CAA43171	TDR6 (*Solanum lycopersicum*)	4e-123
LE22O19	SGN-U580463	−2.148	P05118	Wound-induced proteinase inhibitor 1	1e-56
LE32K23	SGN-U568929	−2.269	CAA43171	TDR6 (*Solanum lycopersicum*)	4e-123
LE5A07	SGN-U579381	−2.643	BAA94287	MADS-box protein pMADS4 (Petunia x hybrida)	7e-108
LE27C20	SGN-U591985	−2.742	O22456	Developmental protein SEPALLATA 3	4e-28
LE7J23	SGN-U577905	−2.835	AF448521	*Lycopersicon esculentum* MADS-box transcription factor MADS-MC (MADS-MC)	1e-124
LE32I09	SGN-U577258	−2.966	AAA80496	Flower-specific gamma-thionin-like protein (*Solanum lycopersicum*)	3e-37
LE8D20	SGN-U581481	−5.326	CAC83066	MADS-box protein TM29 (*Solanum lycopersicum*)	6e-123
LE31P14	SGN-U577283	−5.681	NP_200507	Jasmonic-induced protein 21 (JIP21) (*Solanum lycopersicum*)	2e-118
LE15K15	SGN-U585985	−5.742	Q9LTS3	Cytokinin dehydrogenase 3 (CKX3) (*Arabidopsis thaliana*)	5e-48
LE26B03	SGN-U572646	−9.556	AAW83046	CRABS CLAW (*Nicotiana tabacum*)	1e-65
LE1F12	SGN-U569398	−13.416	P48007	Floral homeotic protein PISTILLATA (GLO1)	4e-52

All together, these results reveal that *UFD* may particularly regulate genes coding for transcription factors required for floral organ development and genes involved in hormone signaling (Figure 3). Some of these genes were investigated by qRT-PCR in order to confirm microarray expression data and to better characterize the *UFD* function.

### Expression of genes involved in floral organ identity and growth

**Figure 4:**
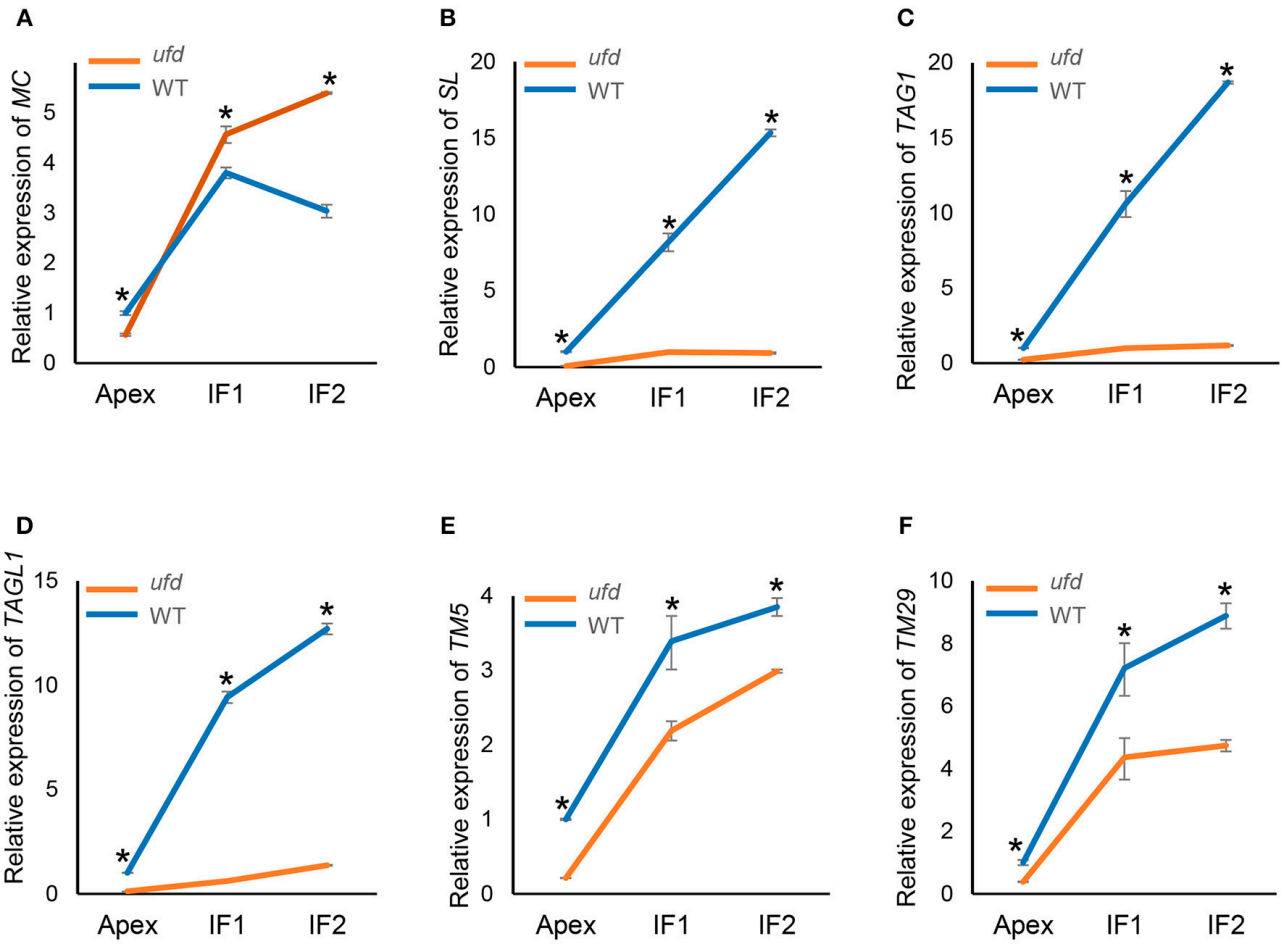
**Quantitative real-time PCR determination of expression of (A)** *MACROCALYX (MC)*, **(B)** *STAMENLESS (SL)*, **(C)** *TOMATO AGAMOUS 1 (TAG1)*, **(D)** *TOMATO AGAMOUS-LIKE 1 (TAGL1)*, **(E)** *TOMATO MADS BOX 5 (TM5)*, and **(F)** *TOMATO MADS BOX 29 (TM29)* in the wild type (WT) and the *unfinished flower development* (*ufd*) mutant flowering inflorescences along their development. Inflorescence developmental stages (i.e., Apex, IF1, and IF2) are described in Material and Methods Section. Error bars show the standard deviation of three independent biological replicates; ^*^significant differences at *P* < 0.05. Panel **(A)** is from Poyatos-Pertíñez et al. (2016).

The expression of floral organ identity genes *MC* (A-class), *SL* (B-class), *TAG1*, and *TAGL1* (C-class) and *TM5*, and *TM29* (E-class) were analyzed by qRT-PCR in *ufd* mutant inflorescences containing floral buds at increasing developmental stages (Figure 4). At early stages of flower development *MC* transcript abundance was slightly lower in the *ufd* mutant compared to WT (Figure 4A). Later, expression of *MC* increased significantly from flowering shoot apex stage in both WT and *ufd*, although it was down-regulated in late wild type inflorescences (IF2 stage) but continued to increase in *ufd* inflorescences (Figure 4A). In wild type plants, expression of the floral identity genes *SL, TAG1*, and *TAGL1* increased as the inflorescence developed, while in the *ufd* plants, these genes were strongly repressed independently of the inflorescence developmental stage (Figures 4B–D). Thus, microarray observations were confirmed since a reduced expression of the B- and C-class genes was found in the *ufd* mutant compared to the WT. Similarly, *TM5* and *TM29*, whose transcript levels increased during the floral development, were significantly down-regulated in *ufd* inflorescences (Figures 4E,F).

**Figure 5:**
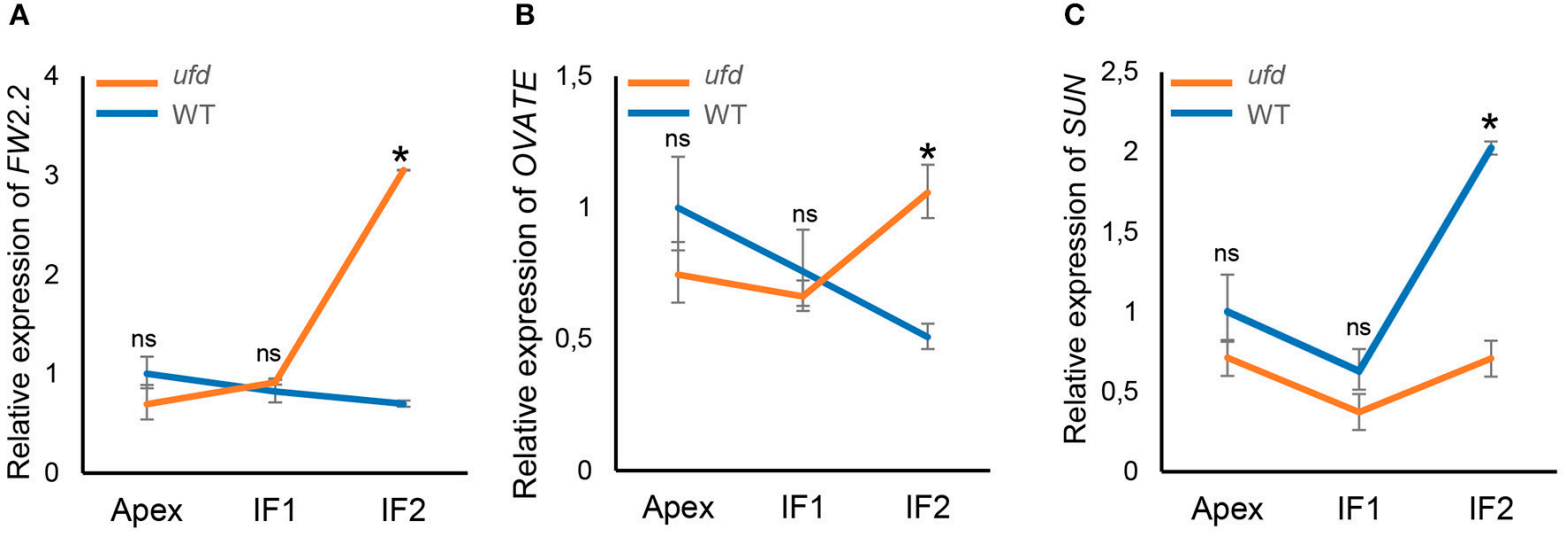
**Quantitative real-time PCR determination of expression of (A)** *FRUIT WEIGHT 2.2 (FW2.2)*, **(B)** *OVATE* **(C)**, and *SUN* in the wild type (WT) and the *unfinished flower development* (*ufd*) mutant flowering inflorescences along their development. Inflorescence developmental stages (i.e., Apex, IF1, and IF2) are described in Material and Methods Section. Error bars show the standard deviation of three independent biological replicates; ns, no statistically significant differences, ^*^ significant differences at *P* < 0.05.

Likewise, regulatory genes *FW2.2, OVATE*, and *SUN*, all of them involved in floral organ growth, were analyzed by qRT-PCR (Figure 5). Non-significance differences between WT and *ufd* were found for these three genes at the apex and young inflorescence (IF1) stages; however, they were differentially expressed at IF2 stage (*P* < 0.05). Thereby, the negative regulators of cell division *FW2.2* and *OVATE* were both overexpressed in *ufd* compared with wild type plants (Figures 5A,B), whereas the expression of *SUN*, a positive regulator of organ growth, was repressed in the *ufd* mutant plants (Figure 5C).

### Endogenous hormone concentration in the *ufd* mutant

**Figure 6:**
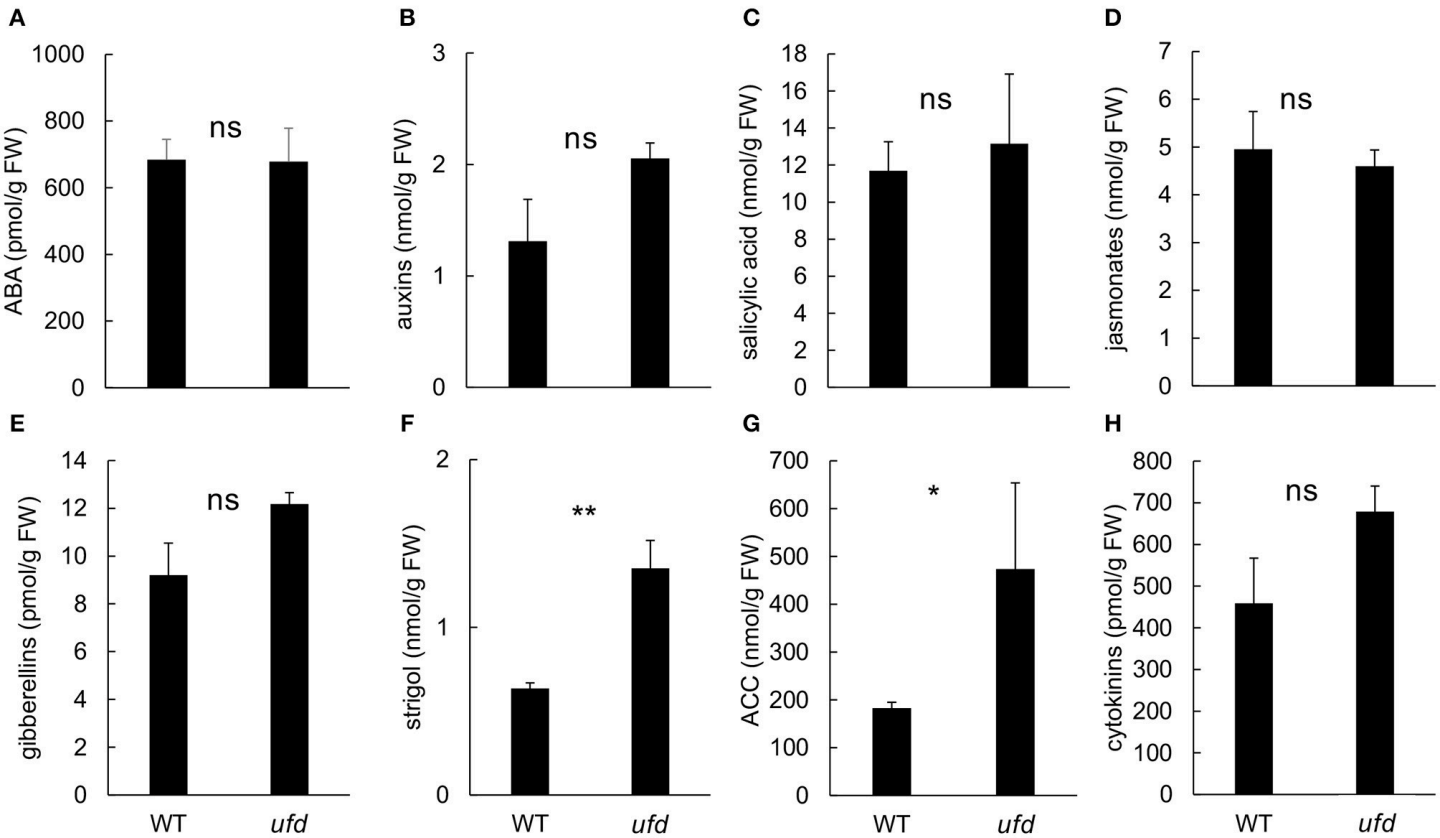
**Impact of the ***unfinished flower development (ufd)*** mutation on the endogenous hormone content of flowering shoot apices. (A)** Total abscisic acid (ABA). **(B)** Total auxins. **(C)** Salicylic acid. **(D)** Total jasmonates. **(E)** Total gibberellins. **(F)** Strigol. **(G)** Ethylene precursor 1-aminocyclopropane-1-carboxylic acid (ACC). **(H)** Total cytokinins. Error bars show the standard deviation of three independent biological replicates; ns, no statistically significant differences, ^*^significant differences at *P* < 0.05, ^**^significant differences at *P* < 0.01. Details of the different forms inside phytohormone groups are presented in Supplementary Table 6.

Microarray results indicated that *ufd* mutation affected transcriptional activity of genes involved in hormone signaling. In order to check if hormone content was also altered in *ufd* mutant plants, we investigated the hormonal profile in *ufd* flowering shoot apices. The *ufd* mutation did not affect the abscisic acid (Figure 6A), salicylic acid (Figure 6C), the jasmonate (Figure 6D) and cytokinins (Figure 6H) concentrations of tomato apices. The strigol (Figure 6F) and ACC (Figure 6G) concentrations were higher in the apices of the *ufd* mutant compared to the wild type. Differences between genotypes also depended on the hormone forms. Regarding the auxins, no significant differences between genotypes were observed for the total auxin concentration (Figure 6B) but free indole-3-acetic acid (IAA, 128 ± 14 vs. 72 ± 9 pmol/g) and conjugated IAA (15 ± 2 vs. 8 ± 0.4 pmol/g and 2.8 ± 0.2 vs. 1.1 ± 0.1 pmol/g for IAA-aspartate and IAA-glutamate, respectively) were detected at higher concentration in *ufd* than wild type flowering apices (*P* < 0.05; Supplementary Table 6). On the same way, total gibberellin content did not differ between genotypes (Figure 6E) but the concentration of GA19 was higher in the *ufd* mutant (7.4 ± 0.4 vs. 5.7 ± 0.4 pmol/g, *P* < 0.05; Supplementary Table 6). At the cytokinins level, the main significant differences between genotypes were observed for the phosphate forms of trans-zeatin (trans-zeatin riboside monophosphate, *P* < 0.0001) and cis-zeatin (cis-zeatin riboside monophosphate, *P* < 0.001), which were respectively more than three (11.0 ± 1.2 vs. 3.4 ± 0.6 pmol/g) and two times (2.7 ± 0.2 vs. 1.2 ± 0.3 pmol/g) higher in the *ufd* mutant compared to WT (Supplementary Table 6).

## Discussion

### The *ufd* mutation affects floral organ growth

The tomato *ufd* mutant has been identified by screening an EMS-mutagenized M2 population of tomato (cv. Moneymaker), and further characterized in this study. This mutant shows a singular phenotype that affects the growth of floral organs, in such way that they are unable to complete their development. Consequently, flower development is blocked at early stage (stage 5–6 according to Brukhin et al., 2003), when all floral organ primordia have been initiated in their appropriate whorls. The screening of a large segregating population proved that the *ufd* mutation is inherited as recessive and it does not affect other vegetative or reproductive traits. The *UFD* function is thus restricted to floral organogenesis, a developmental process where *UFD* is crucial for making fully developed floral organs, although it is not required for the proper initiation of floral organ primordia.

Mutations in genes affecting floral organ identity and growth usually resulted in homeotic conversion of floral whorls or in abnormal development of the floral organs but did not stop their development at primordia stage (for a review, see Lozano et al., 2009; Causier et al., 2010; Mizzotti et al., 2014; Lombardo and Yoshida, 2015). Even a total loss of floral organ identity in Arabidopsis flowers resulted in the conversion of sepals, petals, stamens, and carpels in leaf-like organs as observed in the *sep1-4* quadruple mutant or in mutants combining mutations in all floral homeotic genes (Bowman et al., 1991; Pelaz et al., 2000; Ditta et al., 2004). In tomato, floral organ identity was partially or completely lost when representative genes of MADS-box classes A (*MC)*, B (*SL, TM6, TPI* and *TPIB*), C (*TAG1*), and E (*TM5* and *TM29*) were mutated or down-regulated (Lozano et al., 2009; Geuten and Irish, 2010; Quinet et al., 2014; Yuste-Lisbona et al., 2016), promoting homeotic changes in the floral organs where these gene functions are required. Additional floral whorls or floral organs were also observed in antisense *TM5* plants and ectopic shoots with partially developed leaves and secondary flowers emerged from the fruit in antisense *TM29* plants (Pnueli et al., 1994b; Ampomah-Dwamena et al., 2002). Furthermore, tomato mutations that altered regulatory genes involved in the activity and size of the FM as *INFLORESCENCE MERISTEM ACTIVITY* and the tomato homologous to *CLAVATA* genes have recently been isolated and further characterized (Sicard et al., 2008; Xu et al., 2015). Once again, the corresponding mutations affected inflorescence architecture and the number of floral organs, although alterations in floral organ identity and growth were not reported. To our knowledge, no other single mutants similar to *ufd* have been previously reported in tomato or in other model plant species such as Arabidopsis, Antirrhinum, and rice. Only the flowers of the Arabidopsis *arf6 arf8* double mutant showed a similar phenotype to the *ufd* mutant. Thus, *arf6* and *arf8* single mutants displayed a delay in both stamen filament elongation and anther dehiscence, which reduced self- pollination, whereas floral organ growth was arrested in *arf6 arf8* double mutant resulting in the formation of infertile closed buds (Nagpal et al., 2005). However, *arf6 arf8* floral buds were blocked after carpel primordia were initiated, at a later developmental stage compared to *ufd* floral buds (Figure 2H). Therefore, it suggests that characterization of *ufd* mutation could reveal a regulatory gene specifically required for the normal growth of floral organs in tomato. Hopefully, the cloning of *UFD* gene, which is currently underway, will shed light on its function during flower development.

### *UFD* affects the expression of floral organ identity and growth genes during tomato flower development

It is generally accepted that once the FM has been specified, FM identity genes trigger floral organ identity genes, which in turn determine floral organ patterning according to the ABC(DE) model (reviewed in Ó'Maoiléidigh et al., 2014; Wellmer et al., 2014). However, after floral organ primordia are initiated, identity, and growth of differentiated cells must be specifically coordinated to promote a full development of floral organs. Expression levels of ABC genes are slightly lower in *ufd* mutant plants at the onset of flower development, but later during flower development B- and C-class genes were significantly repressed. Previous reports have proved that the ABC genes play pivotal roles not only determining floral organ identity at early stages of flower development but they are expressed during late stages of floral organogenesis indicating that their functions are needed throughout floral development. Moreover, several pieces of evidence have highlighted the close link between the identity and the final size of floral organs at the end stages of flower development (reviewed in Dornelas et al., 2011; Wellmer et al., 2014). However, genetic and molecular mechanisms coordinating differentiation and growth of specific cells that constitute floral organs still remain unknown (Dornelas et al., 2011). Results reported in the present study suggest that *UFD* gene may be involved in the genetic network linking both developmental processes in tomato. Transcriptomic and expression analyses performed in *ufd* mutant showed that the expression levels of ABC identity genes were smaller than those observed in WT plants during late stages of flower development, suggesting that floral organ primordia, which are unable to grow and complete their development, do not maintain their identity. Furthermore, the failure to grow floral organ primordia in *ufd* plants is supported by the up-regulation of genes involved in the repression of cell division, as *FW2.2* and *OVATE*. Accordingly, *SUN* is down-regulated in *ufd* inflorescences as expected from a positive regulator of floral organ growth. Overall, the results obtained suggested that identity and growth are parts of the same developmental program leading to floral organ formation, and that *UFD* may regulate both ABC and cell division genes. It is also reasonable to think that organ cells have lost their identity due to their incapacity to proliferate and grow, and therefore, *UFD* could be a target of ABC identity genes. Under this genetic scenario, *UFD* would participate in a positive feed-back regulatory pathway enabling the maintenance of the identity of a given floral organ and the differentiation and growth of specific cells composing this organ. Such a hypothesis would be in accordance with the autoregulatory feedback mechanism, which involves MADS-box and other interacting transcription factors (Dornelas et al., 2011). However, other hierarchical relationships between *UFD* and organ identity genes cannot be discarded until *UFD* is cloned and its functional role in establishing floral organ identity is further determined.

Expression results proved that *UFD* gene is required for the transcriptional activity of the B-, C-, and E-class genes leading to further development of the floral organ primordia mainly in the three inner whorls. Interestingly, expression of *MC* in *ufd* floral buds did not differ significantly from that of WT up until the late stages of inflorescence development, when *MC* was up-regulated. In addition, sepals of *ufd* floral buds displayed a normal development and appeared to be more elongated than the remaining floral organs when they were initiated, however their growth was also arrested at the beginning of floral organogenesis. Sepals were suggested as the default state of floral organs and it was proposed that the activities of the FM identity genes during the specification of floral meristems could be involved in specifying sepal identity (Causier et al., 2010; Wellmer et al., 2014). It is true that the first whorl of tomato flowers seems to have a special status since mutations in *MC* only change sepal identity (Vrebalov et al., 2002; Yuste-Lisbona et al., 2016) while mutations of B- and C-class genes affect floral organs of two consecutive floral whorls and loss-of-function of E-class genes alter the three inner whorls (Pnueli et al., 1994b; Ampomah-Dwamena et al., 2002). In this context, *UFD* expression should be differently regulated in sepals and in the three innermost floral whorls. Our qRT-PCR results confirmed that *MC* expression was observed in *ufd* mutant while B-, C-, and E-class genes remained down-regulated during inflorescence and flower development. Moreover, *MC* expression continued to increase during inflorescence development in the *ufd* mutant while it decreased in the WT. That *UFD* represses the *MC* expression during floral organ development and activates the B- and C-class genes for proper development of petals, stamens, and carpels should not be excluded.

### *UFD* participates in the hormone signaling pathways involved in floral development

The interaction among different phytohormones is thought to mediate the formation of organ primordia and the differentiation of floral organs (Chandler, 2011; Wellmer et al., 2014). In accordance, transcriptomic analyses in Arabidopsis revealed that genes involved in FM and floral organ identity as well as in cell division regulate the expression of many genes involved in hormone responses and metabolism (Kaufmann et al., 2009, 2010; Yant et al., 2010; Wuest et al., 2012; Ó'Maoiléidigh et al., 2013; Winter et al., 2015). In the same way, our results on transcriptome profile showed that genes related to jasmonic acid, cytokinin, and auxin signaling were down-regulated in *ufd* mutant inflorescences while genes involved in ethylene response were up-regulated suggesting that hormone response could be mediated by *UFD* transcriptional activity. In tomato plants, insensitivity to hormones often resulted in floral organ defects. Jasmonic acid insensitivity leads to a female sterile phenotype and defects in stamen development in the *jai1-1* mutant (Li et al., 2004). This phenotype is partly due to a premature expression of ethylene signaling regulators showing that jasmonic acid and ethylene could have an antagonist effect during flower development (Dobritzsch et al., 2015). Down-regulation of *ARF6* and *ARF8* resulted in shortened petals, stamens and styles and female sterility (Liu et al., 2014), some of these phenotypes being also attributed to reduced jasmonate production or signaling (Nagpal et al., 2005). Nevertheless, the way in which floral transcription factors and hormones interact to control floral organ growth remained largely unknown. Recently, it has been suggested that *SEPALLATA 3* (*SEP3*) could regulate floral organ development by modulating auxin response in Arabidopsis (Kaufmann et al., 2009). *SEP3* belongs to SEP subfamily of MADS-box genes needed to specify the identity of FM and floral organs through the formation of multimeric complexes with ABC proteins (Immink et al., 2009). Therefore, *SEP3* could be a linking factor between identity and hormonal signals required for floral organ growth. The fact that expression of *TM29*, a tomato member of *SEP* family, is severely repressed in *UFD* would support this hypothesis.

Moreover, our results showed that the *ufd* mutation not only affects the expression of genes involved in hormone response but also affects the inflorescence hormonal profile. The production of ACC, strigol, and some forms of auxins, gibberellins, and cytokinins were increased in *ufd* compared to WT. Modification of the hormonal profile was previously reported in B-class tomato mutants *sl* and *sl-2* (Sawhney, 1974; Singh et al., 1992; Singh and Sawhney, 1998, Quinet et al., 2014). These observations might indicate that hormones play a role in proper development of floral organs. In Arabidopsis, the initiation and outgrowth of floral primordia and floral organ initiation depend on the activity of several phytohormones, mainly auxins (Chandler, 2011; Wellmer et al., 2014). Cytokinins and gibberellins also interact with auxins at this level, e.g., in the determination of boundaries between floral organ primordia (Ding et al., 2015). Involvement of these hormones at the early stage of flower development could explain the higher concentration of IAA and some cytokinin forms in the *ufd* mutant flowering apices. Strigolactones were recently shown to play a hormonal role in plant development (de Saint Germain et al., 2013) and the high strigol concentration in the *ufd* mutant flowering shoot apex suggests a potential role in flower development in tomato. Further investigations will be required to unravel the hormonal crosstalk underlying floral organ growth in tomato and how it interacts with *UFD*.

Summarizing, *UFD* is a regulator of the tomato floral organogenesis that ensures the proper development of floral organ primordia by affecting the expression of ABC(DE) transcription factors and genes involved in cell division and hormone pathways.

## Author contributions

SP and RL were involved in experimental design and interpretation of data. SP, MQ, AO, and EG. performed experiments and analyzed data; SP, MQ, FY, and RL wrote, reviewed and edited the manuscript; CP and AG supported microarray analysis and data handling; JC and TA supervised experiments and provided critical review and editing of the manuscript. All authors contributed to editing and approving the final version of the manuscript.

## Funding

This research was supported by the Spanish Ministry of Economy and Competitiveness and the EU European Regional Development Fund (Grants BIO2009-11484 and AGL2015-64991-C3-R-1). We also thank Campus de Excelencia Internacional Agroalimentario (CeiA3) for providing financial support. AO is a recipient of a PhD fellowship from the Ministerio de Ciencia e Innovación of Spain (BIO2009-11484).

### Conflict of interest statement

The authors declare that the research was conducted in the absence of any commercial or financial relationships that could be construed as a potential conflict of interest.
