# Supplementary material for: A Factor Linking Floral Organ Identity and Growth Revealed by Characterization of the Tomato Mutant unfinished flower development (ufd)
Source: Front Plant Sci. 2016 Nov 7;7:1648. doi: 10.3389/fpls.2016.01648 (PMC5098122; doi:10.3389/fpls.2016.01648)
Supplement: Supplementary file 1 [file Table1.PDF]

Supplementary Table 1 Selected primers used for qRT-PCR analysis

Target gene	Primers	Sequence (5´to 3´)	NCBI identifier
UBI3	Forward	CCAAGATCCAGGACAAGGAA	X58253.1
	Reverse	AAATCAAACGCTGCTGGTCT	
MC	Forward	TCTCTGAACTGCAGAAAAAGGA	NM_001247736.1
	Reverse	TAGTTTGCTGGTGCCATTCA	
SL	Forward	ACTTACGCCTTCAACCCAAC	DQ674532.1
	Reverse	ATCAGAGCCACCTCCACTGT	
TAGL1	Forward	AAAAGAGGGAGATTGAGCTGC	NM_001313930.1
	Reverse	CTCTACCTCTGCTATCTTTGCG	
TAG1	Forward	CTTGATGCCAGGGAGTTCAT	NM_001308296.2
	Reverse	ATCGAATTGCTGAGGTGGAG	
TM5	Forward	AGTAGGAGGAGCAGGGCCTA	NM_001247455.2
	Reverse	CCGTTAGGTCCAGGATGAAA	
TM29	Forward	GTCAGCAGCAACATCCTCAA	NM_001246982.2
	Reverse	CATTCACAGCATCCAACCAG	
FW2.2	Forward	TATGGTGTTGGGTTGCCTCT	HG975514.1
	Reverse	CATGCACAAACACCTTGAGC	
OVATE	Forward	TTGAAGCTTCGGTTCTCCAG	NM_001247292.2
	Reverse	AGGAGGTCGTAAGGCCAAAT	
SUN	Forward	TCCCTGTTTGTTTTCCCTTG	XM_004249940.2
	Reverse	AAATGCTTCCAGGTGAATGG	
