# Supplementary material for: A Factor Linking Floral Organ Identity and Growth Revealed by Characterization of the Tomato Mutant unfinished flower development (ufd)
Source: Front Plant Sci. 2016 Nov 7;7:1648. doi: 10.3389/fpls.2016.01648 (PMC5098122; doi:10.3389/fpls.2016.01648)
Supplement: Supplementary file 2 [file Table2.PDF]

Supplementary Table 2 Comparison of *unfinished flower development* (*ufd*) and wild type plant flowering time in standard conditions. A total of 543 plants, 391 wild-type and 152 *ufd* mutants, were screened in the M3 generation. Means value ± SD. No significant differences were found according to the t-test ($P < 0.001$)

Trait	Phenotype at the *UFD* locus	
	ufd	**wild-type**
No. of leaves to the first inflorescence	6.21 ± 0.16	6.37 ± 0.34
No. of leaves between the first and second inflorescence	3.50 ± 0.71	3.70 ± 0.29
No. of leaves between the second and third inflorescence	3.00 ± 0.00	3.16 ± 0.45
