# Supplementary material for: A Factor Linking Floral Organ Identity and Growth Revealed by Characterization of the Tomato Mutant unfinished flower development (ufd)
Source: Front Plant Sci. 2016 Nov 7;7:1648. doi: 10.3389/fpls.2016.01648 (PMC5098122; doi:10.3389/fpls.2016.01648)
Supplement: Supplementary file 3 [file Table3.PDF]

Supplementary Table 3 Gene ontology analysis of differentially expressed genes up-regulated in the *unfinished flower development* (*ufd*) mutant

**Gene Ontology term**	**Cluster frequency**	**Genome frequency of use**	**P-value***
<u>Cellular component</u>			
Cell	150 out of 223 genes, 67.3%	6848 out of 12672 genes, 54.0%	0.00416
Intracellular	139 out of 223 genes, 62.3%	5808 out of 12672 genes, 45.8%	0.0000518
Organelle	127 out of 223 genes, 57.0%	5047 out of 12672 genes, 39.8%	0.000017
Membrane-bounded organelle	121 out of 223 genes, 54.3%	4879 out of 12672 genes, 38.5%	0.00013
Cytoplasm	112 out of 223 genes, 50.2%	4474 out of 12672 genes, 35.3%	0.00034
Nucleus	66 out of 223 genes, 29.6%	2276 out of 12672 genes, 18.0%	0.00151
Non-membrane-bounded organelle	44 out of 223 genes, 19.7%	863 out of 12672 genes, 6.8%	1.26E-08
Macromolecular complex	43 out of 223 genes, 19.3%	1332 out of 12672 genes, 10.5%	0.00702
Cytosol	36 out of 223 genes, 16.1%	924 out of 12672 genes, 7.3%	0.00059
Ribonucleoprotein complex	34 out of 223 genes, 15.2%	490 out of 12672 genes, 3.9%	6.9E-10
Ribosome	31 out of 223 genes, 13.9%	364 out of 12672 genes, 2.9%	3.14E-11
Nucleolus	26 out of 223 genes, 11.7%	394 out of 12672 genes, 3.1%	0.000000768
Vacuole	24 out of 223 genes, 10.8%	630 out of 12672 genes, 5.0%	0.0365
Cytosolic ribosome	24 out of 223 genes, 10.8%	267 out of 12672 genes, 2.1%	6.12E-09
Ribosomal subunit	22 out of 223 genes, 9.9%	188 out of 12672 genes, 1.5%	2.18E-10
Mitochondrial intermembrane space	4 out of 223 genes, 1.8%	15 out of 12672 genes, 0.1%	0.01312
<u>Molecular function</u>			
Nucleic acid binding	45 out of 223 genes, 20.2%	1497 out of 12672 genes, 11.8%	0.031
Structural constituent of ribosome	27 out of 223 genes, 12.1%	250 out of 12672 genes, 2.0%	0.000
Structural molecule activity	27 out of 223 genes, 12.1%	291 out of 12672 genes, 2.3%	0.000
RNA binding	18 out of 223 genes, 8.1%	370 out of 12672 genes, 2.9%	0.015
rRNA binding	7 out of 223 genes, 3.1%	58 out of 12672 genes, 0.5%	0.010
Chitinase activity	4 out of 223 genes, 1.8%	17 out of 12672 genes, 0.1%	0.028
Adenylate kinase activity	3 out of 223 genes, 1.3%	8 out of 12672 genes, 0.1%	0.043

* P-value corrected by Bonferroni method.
