# Supplementary material for: A Factor Linking Floral Organ Identity and Growth Revealed by Characterization of the Tomato Mutant unfinished flower development (ufd)
Source: Front Plant Sci. 2016 Nov 7;7:1648. doi: 10.3389/fpls.2016.01648 (PMC5098122; doi:10.3389/fpls.2016.01648)
Supplement: Supplementary file 4 [file Table4.PDF]

Supplementary Table 4 Gene ontology analysis of differentially expressed genes down-regulated in the *unfinished flower development* (*ufd*) mutant

Gene Ontology term	Cluster frequency	Genome frequency of use	P-value*
Cellular component			
Photosystem	7 out of 102 genes, 6.9%	86 out of 12672 genes, 0.7%	0.00042
Photosystem II	6 out of 102 genes, 5.9%	68 out of 12672 genes, 0.5%	0.00126
Photosystem I	6 out of 102 genes, 5.9%	58 out of 12672 genes, 0.5%	0.0005
Light-harvesting complex	2 out of 102 genes, 2.0%	4 out of 12672 genes, 0.0%	0.02818
Thylakoid light-harvesting complex	2 out of 102 genes, 2.0%	4 out of 12672 genes, 0.0%	0.02818
PSII associated light-harvesting complex II	2 out of 102 genes, 2.0%	3 out of 12672 genes, 0.0%	0.01416
Photosystem II antenna complex	2 out of 102 genes, 2.0%	2 out of 12672 genes, 0.0%	0.00474
Molecular funtion			
Transcription regulator activity	15 out of 102 genes, 14.7%	717 out of 12672 genes, 5.7%	0.04841
Transcription factor activity	13 out of 102 genes, 12.7%	446 out of 12672 genes, 3.5%	0.00472
Sequence-specific DNA binding	11 out of 102 genes, 10.8%	291 out of 12672 genes, 2.3%	0.00177
Chlorophyll binding	5 out of 102 genes, 4.9%	45 out of 12672 genes, 0.4%	0.00237
Serine-type endopeptidase inhibitor activity	4 out of 102 genes, 3.9%	28 out of 12672 genes, 0.2%	0.00572
Endopeptidase inhibitor activity	4 out of 102 genes, 3.9%	42 out of 12672 genes, 0.3%	0.02871
Protease inhibitor activity	4 out of 102 genes, 3.9%	42 out of 12672 genes, 0.3%	0.02871

* P-value corrected by Bonferroni method.
