# Supplementary material for: A Factor Linking Floral Organ Identity and Growth Revealed by Characterization of the Tomato Mutant unfinished flower development (ufd)
Source: Front Plant Sci. 2016 Nov 7;7:1648. doi: 10.3389/fpls.2016.01648 (PMC5098122; doi:10.3389/fpls.2016.01648)
Supplement: Supplementary file 5 [file Table5.PDF]

**Supplementary Table 5** Hierarchical clusterring of differentially expressed genes in *unfinished flower development* (*ufd*) mutant relative to wild type plants ($P < 0.05$)

| Clusters | Probe ID | Gene ID | Fold Change | GenBank ID | Annotation | e-value |
|---|---|---|---|---|---|---|
| Cluster 1 | LE29C09 | SGN-U563323 | 3.341 | AF011555 | LEJA2 Jasmonic acid 2 | 0 |
| | LE17B14 | SGN-U571844 | 2.702 | AAA65637 | Peroxidase | 0 |
| | LE11A11 | SGN-U578977 | 1.194 | AAR83884 | Ly200 protein [Capsicum annuum] | 2e-060 |
| | LE9D23 | SGN-U589805 | 4.206 | CAO39940 | Unknown protein | 2e-19 |
| | LE9L09 | SGN-U579357 | 1.752 | P32111 | Probable glutathione S-transferase (Pathogenesis-related protein 1) | 1e-104 |
| | LE6G19 | SGN-U580000 | 3.867 | AAG16757 | Putative glutathione S-transferase T2 [Lycopersicon esculentum] | 1e-117 |
| | LE9L20 | SGN-U585247 | 1.325 | BAE48664 | Pectate lyase family protein | 0 |
| | LE26P24 | SGN-U580191 | 8.125 | P17786 | Elongation factor 1-alpha | 0 |
| | LE24P06 | SGN-U579551 | 1.556 | Q05538 | Basic endochitinase | 7e-169 |
| | LE21O10 | SGN-U578255 | 1.136 | ABB16983 | 60S ribosomal protein L5 (RPL5B) | 2e-122 |
| | LE6D02 | SGN-U579010 | 1.268 | ABB29928 | 60S ribosomal protein L18A (RPL18aC) | 2e-92 |
| | LE23F22 | SGN-U578883 | 1.923 | CAA58151 | 2-oxoglutarate-dependent dioxygenase | 1e-114 |
| | LE14E03 | SGN-U588273 | 1.653 | ACK57683 | Peroxidase | 1e-73 |
| | LE12H19 | SGN-U578857 | 1.146 | AAR83877 | 60S ribosomal protein L19 [Capsicum annuum] | 2e-095 |
| | LE3C19 | SGN-U580607 | 1.183 | ABA40467 | Glycoprotein-like protein [Solanum tuberosum] | 7e-67 |
| | LE14C03 | SGN-U577299 | 1.256 | P49209 | 60S ribosomal protein L9 (RPL90A/C) similar to RIBOSOMAL PROTEIN L9 [Arabidopsis thaliana] | 3e-92 |
| | LE27P24 | SGN-U580274 | 1.073 | CAO49446 | Unknown protein | 1e-117 |
| | LE17F23 | SGN-U577821 | 2.225 | Q05047 | Cytochrome P450 [Catharanthus roseus] | 4e-130 |
| | LE3M22 | SGN-U577973 | 1.154 | P51413 | 60S ribosomal protein L17 (RPL17A) [Arabidopsis thaliana] | 4e-78 |
| | LE24L17 | SGN-U581140 | 1.183 | Q8HSW1 | Adenylate kinase, chloroplast precursor (ATP-AMP transphosphorylase) | 2e-129 |
| | LE12M11 | SGN-U584696 | 1.096 | CAO21574 | Unknown protein | 6e-88 |
| | LE14G06 | SGN-U572877 | 1.089 | ABK42076 | 26S proteasome subunit RPN7 [Capsicum annuum] | 0 |
| | LE21O22 | SGN-U583016 | 1.923 | BAA89235 | WRKY-type DNA binding protein, TMV response-related [Nicotiana tabacum] | 3e-99 |
| | LE18E04 | SGN-U583014 | 2.020 | BAA89235 | WRKY-type DNA binding protein, TMV response-related [Nicotiana tabacum] | 4e-89 |
| | LE4L11 | SGN-U566414 | 1.133 | BAC23035 | 26S proteasome AAA-ATPase subunit RPT4a [Solanum tuberosum] | 0 |
| | LE21E04 | SGN-U578627 | 1.146 | Q9XGM1 | Vacuolar ATP Synthase Subunit D [Arabidopsis thaliana] | 4e-114 |
| | LE18M07 | SGN-U585573 | 1.089 | AAD04946 | Expressed protein contains similarity to PrMC3 [Pinus radiata] | 1e-112 |
| | LE33D05 | SGN-U575698 | 1.118 | AEE29192 | Endoxyloglucan transferase A2 [Arabidopsis thaliana] | 2e-27 |
| | LE8P03 | SGN-U563149 | 1.150 | AAX63403 | Flavonoid 3-glucosyl transferase [Solanum tuberosum] | 0 |
| | LE30I06 | SGN-U595080 | 1.460 | AAL54887 | Cytochrome P450-dependent fatty acid hydroxylase [Nicotiana tabacum] | 1e-40 |
| | LE33B21 | SGN-U573661 | 1.047 | Q43082 | Porphobilinogen deaminase, chloroplast precursor (PBG) (Hydroxymethylbilane synthase) (HMBS) (Pre-uroporphyrinogen synthase) | 6e-150 |
| | LE6N12 | SGN-U580909 | 1.674 | O49954 | Glycine dehydrogenase [decarboxylating], mitochondrial precursor (Glycine decarboxylase) (Glycine cleavage system P-protein) | 2e-73 |
| | LE26A07 | SGN-U579236 | 2.922 | BAD98961 | 2-oxoglutarate-dependent dioxygenase [Solanum lycopersicum] | 0 |
| | LE19G07 | SGN-U580587 | 1.206 | AAF60293 | Chaperonin 21 precursor [Lycopersicon esculentum] | 2e-110 |
| | LE29C10 | SGN-U564094 | 1.115 | AAO44077 | Adenylate kinase, putative, similar to adenylate kinase (ATP-AMP transphosphorylase)[Arabidopsis thaliana] | 3e-115 |
| | LE23E09 | SGN-U573749 | 1.097 | P92792 | Mitochondrial import receptor subunit TOM20 | 5e-77 |
| | LE15A21 | SGN-U573117 | 1.075 | ABU49721 | WRKY transcription factor 2 [Solanum tuberosum] | 2e-154 |
| | LE7I09 | SGN-U578506 | 3.396 | No hits | No hits | |
| | LE8K02 | SGN-U579967 | 1.149 | CAO46269 | Unknown protein | 7e-39 |
| | LE9L19 | SGN-U567747 | 1.071 | CAA74776 | NAD-dependent isocitrate dehydrogenase [Nicotiana tabacum] | 0 |
| | LE13E18 | SGN-U578062 | 1.162 | CAO68204 | Unknown protein | 4e-118 |
| | LE19E04 | SGN-U581065 | 1.446 | ABK93578 | Unknown protein | 1e-72 |
| | LE11N11 | SGN-U578722 | 1.205 | AAC49972 | ORF; able to induce HR-like lesions [Nicotiana tabacum] | 1e-56 |
| | LE5A06 | SGN-U582597 | 1.256 | BAA76895 | LeArcA1 protein [Solanum lycopersicum] | 6e-178 |
| | LE23F03 | SGN-U582797 | 8.938 | BAA13150 | NT16 polypeptide [Nicotiana tabacum] | 2e-32 |
| | LE1F09 | SGN-U580141 | 1.175 | CAO71457 | Unknown protein | 0 |
| | LE23H21 | SGN-U591643 | 1.173 | AEE83872 | Mo25 family protein [Arabidopsis thaliana] | 2e-29 |
| | LE26K13 | SGN-U573279 | 1.139 | AAG53636 | Eukaryotic translation initiation factor 3G1 | 3e-109 |
| | LE33C08 | SGN-U576586 | 1.086 | AED91940 | GCN5-related N-acetyltransferase | 2e-78 |
| | LE25O09 | SGN-U570718 | 1.319 | AED97611 | Myb transcription factor [Arabidopsis thaliana] | 6e-83 |
| | LE16C06 | SGN-U580857 | 1.521 | AAB08443 | Chitinase, class II [Solanum lycopersicum] | 6e-134 |
| | LE30N08 | SGN-U569435 | 1.298 | BAH20294 | Mannitol dehydrogenase | 9e-113 |
| | LE10E17 | SGN-U583406 | 1.173 | AEE79597 | Nucleolar essential protein-related protein [Arabidopsis thaliana] | 2e-87 |
| | LE6D17 | SGN-U585549 | 1.255 | BAD15365 | Nitrite reductase [Nicotiana tabacum] | 0 |
| | LE15D03 | SGN-U567580 | 1.203 | P46269 | Cytochrome b-c1 complex subunit 8 | 6e-35 |
| | LE4I21 | SGN-U575378 | 1.156 | AAF91282 | DNA-binding protein p24 [Solanum tuberosum] | 7e-129 |
| | LE1K19 | SGN-U577174 | 1.112 | Q8LGE7 | NADH-ubiquinone oxidoreductase 19 kDa subunit (NDUFA8) family protein | 1e-43 |
| | LE24D13 | SGN-U585951 | 1.659 | BAF44192 | SNF1-related kinase [Solanum lycopersicum] | 0 |
| | LE30J22 | SGN-U574873 | 1.919 | AAK95688 | Ethylene-responsive factor 1 [Lycopersicon esculentum] | 4e-92 |
| | LE30B14 | SGN-U583603 | 1.163 | AM236863 | Mitochondrial substrate carrier family protein | 8e-16 |
| | LE18O04 | SGN-U563718 | 1.153 | CAN73938 | Unknown protein | 3e-94 |
| | LE33C13 | SGN-U576561 | 2.351 | NP_189542 | Calcium-binding EF-hand family protein [Arabidopsis thaliana] | 9e-19 |
| | LE7J21 | SGN-U564367 | 1.281 | BAA22813 | Chloroplast nucleoid DNA binding protein [Nicotiana tabacum] | 1e-135 |
| | LE24K05 | SGN-U563958 | 1.105 | BAA22079 | Protein kinase, similar to protein kinase ATMRK1 [Arabidopsis thaliana] | 0 |
| | LE25D18 | SGN-U577356 | 3.057 | No hits | No hits | |
| | LE31L21 | SGN-U576468 | 1.235 | AAC27657 | NaCl-inducible Ca2+-binding protein [Arabidopsis thaliana] | 7e-30 |
| | LE28I16 | SGN-U566888 | 1.091 | AEE86031 | Nucleotide/sugar transporter family protein [Arabidopsis thaliana] | 5e-136 |
| | LE3N19 | SGN-U576121 | 1.947 | NP_197841 | Oxidoreductase, 2OG-Fe(II) oxygenase family protein | 3e-139 |
| | LE5M20 | SGN-U573553 | 1.198 | NP_001067264 | Os12g0613500 | 2e-42 |
| | LE27H07 | SGN-U563528 | 1.084 | AEE82157 | Epoxide hydrolase, putative [Arabidopsis thaliana] | 1e-64 |
| | LE17C04 | SGN-U563851 | 1.865 | AAU93595 | Putative TB2/DP1, HVA22 family protein [Solanum demissum] | 3e-94 |
| | LE31C08 | SGN-U574896 | 1.734 | AEC08624 | GCN5-related N-acetyltransferase (GNAT) family protein [Arabidopsis thaliana] | 2e-48 |
| | LE28J11 | SGN-U585020 | 1.448 | CAB63264 | Calcium-binding protein [Lotus corniculatus var. japonicus] | 6e-60 |
| | LE31O23 | SGN-U583039 | 2.514 | NP_177524 | BEE3, bHLH transcription factor [Arabidopsis thaliana] | 1e-46 |
| | LE27O22 | SGN-U589215 | 1.130 | ABB88703 | Geranyl pyrophosphate synthase [Solanum lycopersicum] | 2e-042 |

| | | | | | |
|---|---|---|---|---|---|
| LE14M10 | SGN-U569469 | 1.287 | CAO66005 | Unknown protein | 3e-49 |
| LE22P09 | SGN-U575082 | 1.089 | AEE74085 | RING finger protein involved in peroxisome biogenesis | 2e-158 |
| LE2C22 | SGN-U582993 | 1.222 | AAC08008 | Type II CPD photolyase PHR1 [Arabidopsis thaliana] | 3e-138 |
| LE28K02 | SGN-U573684 | 1.183 | BAA97375 | Unknown protein | 1e-81 |
| LE2D05 | SGN-U583072 | 1.351 | AAO45726 | TCP protein [Lycopersicon esculentum] | 2e-034 |
| LE13D03 | SGN-U566955 | 1.105 | CAO71025 | Unknown protein | 1e-121 |
| LE11L09 | SGN-U580898 | 1.147 | BAA12064 | RNA-binding protein RZ-1 [Nicotiana sylvestris] | 2e-43 |
| LE22H06 | SGN-U576747 | 1.367 | AEE84367 | FAD-binding domain-containing protein [Arabidopsis thaliana] | 2e-40 |
| LE9P07 | SGN-U565511 | 1.349 | BAG80554 | UDP-glucose:glucosyltransferase [Lycium barbarum] | 2e-40 |
| LE13N10 | SGN-U563566 | 1.246 | ACJ85265 | Unknown protein | 3e-58 |
| LE27I05 | SGN-U568713 | 1.130 | No hits | No hits | |
| LE30J24 | SGN-U572901 | 1.358 | Q8S2T1 | Dehydrodolichyl diphosphate synthase 6 [Arabidopsis thaliana] | 4e-74 |
| LE5I04 | SGN-U570200 | 1.067 | Q8GUK7 | Protein RING membrane-anchor 3 | 3e-16 |
| LE15H10 | SGN-U596545 | 1.095 | AAU29198 | Mitochondrial malate dehydrogenase [Lycopersicon esculentum] | 0 |
| LE25D08 | SGN-U574586 | 1.196 | CAO71215 | Unknown protein | 2e-143 |
| LE29P22 | SGN-U565330 | 1.152 | AED93188 | DNAJ heat shock N-terminal domain-containing protein [Arabidopsis thaliana] | 2e-92 |
| LE3M13 | SGN-U565687 | 1.239 | AEE76205 | AP2/B3-like transcriptional factor family protein [Arabidopsis thaliana] | 6e-30 |

Cluster 2

| | | | | | |
|---|---|---|---|---|---|
| LE25E12 | SGN-U577337 | 1.960 | CAA06997 | Subtilisin-like protease [Solanum lycopersicum] | 0 |
| LE18L09 | SGN-U580535 | 2.033 | ABM06179 | Glutathione transferase, putative | 3e-41 |
| LE4M08 | SGN-U578841 | 3.925 | Q43502 | Proteinase inhibitor type II CEVI57 | 2e-106 |
| LE33C20 | SGN-U580143 | 1.828 | AAL01594 | Pathogenesis-related protein 1b precursor [Solanum tuberosum] | 1e-20 |
| LE10P17 | SGN-U577715 | 1.138 | ABB86257 | 40S ribosomal protein S15-like [Solanum tuberosum] | 3e-80 |
| LE11D16 | SGN-U580698 | 1.101 | AAP80667 | Ribosomal Pr 117 [Triticum aestivum] | 9e-75 |
| LE18M04 | SGN-U579579 | 1.209 | P17093 | RecName: Full=40S ribosomal protein S11 | 4e-69 |
| LE8L16 | SGN-U580580 | 1.177 | ABA46790 | 60S ribosomal protein L13a-like protein [Solanum tuberosum] | 2e-112 |
| LE22P06 | SGN-U571844 | 2.496 | AAA65637 | Peroxidase [Solanum lycopersicum] | 0 |
| LE13F02 | SGN-U583085 | 1.528 | ACK57683 | Peroxidase 4 [Litchi chinensis] | 1e-133 |
| LE17C09 | SGN-U579850 | 2.728 | AEE73926 | Endoribonuclease Dicer-like 2 [Arabidopsis thaliana] | 5e-120 |
| LE14K03 | SGN-U578480 | 1.162 | O22315 | Serine/arginine-rich-splicing factor SR34 | 1e-70 |
| LE23N13 | SGN-U581507 | 1.438 | P19171 | Basic endochitinase B | 1e-74 |
| LE6O08 | SGN-U577557 | 3.269 | AAU95238 | Osmotin-like protein [Solanum phureja] | 4e-156 |
| LE8C19 | SGN-U580303 | 2.710 | NP_849875 | MLP-like protein 28 [Arabidopsis thaliana] | 1e-68 |
| LE28B21 | SGN-U578713 | 1.183 | Q8LB10 | ATP-dependent Clp protease proteolytic subunit-related protein 4 | 2e-111 |
| LE4E13 | SGN-U570976 | 1.176 | ABB87116 | 40S ribosomal protein S19-like [Solanum tuberosum] | 4e-058 |
| LE20O16 | SGN-U567805 | 1.545 | AAB49688 | Wound-induced protein [Lycopersicon esculentum] | 3e-111 |
| LE25G24 | SGN-U584035 | 1.388 | S39507 | Glucuronosyl transferase homolog, ripening-related - tomato (fragment) | 5e-152 |
| LE5N04 | SGN-U567110 | 1.249 | O22797 | Glycolipid transfer protein 1 | 1e-87 |
| LE14N13 | SGN-U577920 | 1.626 | O81833 | G-type lectin S-receptor-like serine/threonine-protein kinase SD1-1 | 7e-100 |
| LE9A14 | SGN-U575371 | 1.174 | AAG43499 | Pyruvate dehydrogenase [Lycopersicon esculentum] | 0 |
| LE18M16 | SGN-U578331 | 1.300 | AEC09286 | 2-oxoglutarate (2OG) and Fe(II)-dependent oxygenase superfamily protein [Arabidopsis thaliana] | 1e-62 |
| LE4A18 | SGN-U580210 | 1.068 | ABI84104 | GTP-binding Rop/Rac GTPase [Petunia inflata] | 7e-102 |
| LE19B03 | SGN-U574885 | 1.183 | AEE83965 | PLATZ transcription factor family protein [Arabidopsis thaliana] | 3e-85 |
| LE25L22 | SGN-U585313 | 1.150 | AEE32058 | YbaK/aminoacyl-tRNA synthetase-associated domain-containing protein [Arabidopsis thaliana] | 4e-96 |
| LE26O24 | SGN-U566850 | 1.170 | O22769 | NADH dehydrogenase [ubiquinone] flavoprotein 2, mitochondrial | 8e-122 |
| LE27N04 | SGN-U574452 | 1.377 | AAR00326 | Rapid alkalinization factor 2 [Solanum chacoense] | 2e-059 |
| LE28L07 | SGN-U577600 | 1.123 | Q9FJH0 | Ras-related protein RABA1f | 9e-110 |
| LE10E05 | SGN-U563916 | 1.399 | NP_191329 | Protein kinase, putative [Arabidopsis thaliana] | 5e-41 |
| LE28P15 | SGN-U567304 | 3.295 | AAK07676 | Non-symbiotic hemoglobin class 1 [Lycopersicon esculentum] | 2e-080 |
| LE15E19 | SGN-U579602 | 1.132 | Q9M339 | 40S ribosomal protein S3-2 | 3e-108 |
| LE24J21 | SGN-U594167 | 1.213 | ABG29113 | Multiprotein bridging factor 1b [Solanum lycopersicum] | 5e-18 |
| LE33P14 | SGN-U569057 | 1.175 | ABR15770 | Putative alcohol dehydrogenase [Solanum lycopersicum] | 0 |
| LE21B11 | SGN-U576432 | 1.235 | AAG10104 | Fibrillarin 2 (FIB2) | 1e-124 |
| LE13C07 | SGN-U576746 | 3.402 | AEC07585 | C2H2 zinc finger protein FZF [Arabidopsis thaliana] | 8e-94 |
| LE15E11 | SGN-U571964 | 8.381 | AAF63515 | TMV-induced protein I [Capsicum annuum] | 2e-71 |
| LE26L14 | SGN-U581493 | 2.697 | No hits | No hits | |
| LE2O12 | SGN-U577327 | 1.221 | Q07761 | 60S ribosomal protein L23a | 3e-46 |
| LE22L17 | SGN-U586454 | 1.831 | O22822 | UDP-glycosyltransferase 74F2 | 2e-149 |
| LE30E03 | SGN-U579144 | 1.054 | AEC08895 | OB-fold nucleic acid binding domain-containing protein [Arabidopsis thaliana] | 2e-037 |
| LE3G12 | SGN-U596360 | 2.003 | NP_174279 | WRKY71; transcription factor [Arabidopsis thaliana] | 1e-37 |
| LE16M06 | SGN-U565284 | 1.183 | AED90842 | Surfeit locus protein 6 [Arabidopsis thaliana] | 3e-32 |
| LE29L16 | SGN-U581957 | 1.218 | AAM63240 | Unknown protein | 2e-81 |
| LE31B21 | SGN-U581771 | 1.157 | Q6DBM8 | Metal tolerance protein B | 6e-101 |
| LE9B11 | SGN-U569156 | 1.180 | ABK95063 | Unknown protein | 2e-50 |
| LE23C18 | SGN-U570847 | 1.213 | Q940U6 | Protein Fluorescent in blue light | 8e-80 |
| LE30D12 | SGN-U571981 | 1.206 | CAA56600 | 36kDA porin II [Solanum tuberosum] | 4e-89 |
| LE25F03 | SGN-U577726 | 1.272 | AAP80667 | Ribosomal Pr 117 [Triticum aestivum] | 2e-75 |
| LE23B10 | SGN-U580500 | 2.428 | BAC23031 | WRKY-type DNA binding protein [Solanum tuberosum] | 3e-82 |
| LE31P05 | SGN-U570189 | 3.304 | AEC08624 | GCN5-related N-acetyltransferase-like protein [Arabidopsis thaliana] | 3e-47 |
| LE7I16 | SGN-U579553 | 1.616 | ABU49723 | WRKY transcription factor 4 [Solanum tuberosum] | 1e-135 |
| LE8O10 | SGN-U564380 | 1.132 | NP_176747 | Small nuclear ribonucleoprotein [Arabidopsis thaliana] | 1e-41 |
| LE19H06 | SGN-U575324 | 1.600 | AAC49208 | Receptor serine/threonine kinase PR5K [Arabidopsis thaliana] | 1e-88 |
| LE27D04 | SGN-U574241 | 1.634 | AAF69542 | F12M16.30 [Arabidopsis thaliana] | 5e-138 |
| LE1B07 | SGN-U568138 | 1.159 | NP_563886 | Unknown protein | 2e-15 |
| LE24K21 | SGN-U564689 | 1.170 | ABK95362 | Unknown protein | 3e-55 |
| LE27J03 | SGN-U570922 | 1.179 | AEE36130 | Ribosomal protein L13 family protein | 3e-78 |
| LE27O23 | SGN-U568817 | 1.198 | AEE74859 | Mitochondrial import inner membrane translocase subunit Tim17/Tim22/Tim23 family protein [Arabidopsis thaliana] | 1e-56 |
| LE12K19 | SGN-U567800 | 1.455 | B9DGU7 | Thiamine pyrophosphokinase 1 | 7e-92 |
| LE33L10 | SGN-U570793 | 1.181 | AAC27035 | Putative ubiquitin activating enzyme E1 [Arabidopsis thaliana] | 0 |

| | | | | | | |
|---|---|---|---|---|---|---|
| | LE9L13 | SGN-U579680 | 30.718 | P10798 | Ribulose bisphosphate carboxylase small chain 3B | 1e-32 |
| | LE24J09 | SGN-U584570 | 1.839 | Q9FK72 | Heat stress transcription factor A-4c | 2e-76 |
| | LE25D20 | SGN-U567621 | 1.667 | AEE82947 | Nuclear transport factor 2 (NTF2) family protein [Arabidopsis thaliana] | 6e-85 |
| | LE14B19 | SGN-U586788 | 1.173 | No hits | No hits | |
| | LE19F15 | SGN-U584944 | 1.127 | AED91219 | Mitochondrial transcription termination factor family protein [Arabidopsis thaliana] | 2e-32 |
| | LE6H18 | SGN-U565967 | 1.213 | Q9SK27 | Early nodulin-like protein 1 OS | 2e-19 |
| | LE16L20 | SGN-U584719 | 1.563 | AEE82626 | Tetratricopeptide repeat domain-containing protein [Arabidopsis thaliana] | 8e-63 |
| | LE16F19 | SGN-U603739 | 1.272 | Q9LHQ7 | mRNA cap guanine-N7 methyltransferase 1 | 5e-19 |
| | LE3I18 | SGN-U581493 | 3.361 | No hits | No hits | |
| | LE14O13 | SGN-U593442 | 4.746 | AAP43673 | PR5-like protein [Lycopersicon esculentum] | 1e-77 |
| | LE14P02 | SGN-U577754 | 1.246 | AAF98368 | Patatin-like protein 1 [Nicotiana tabacum] | 1e-76 |
| Cluster 3 | | | | | | |
| | LE22L06 | SGN-U578451 | 1.143 | AAY97865 | 60S ribosomal protein L10 [Lycopersicon esculentum] | 3e-100 |
| | LE1H15 | SGN-U591112 | 1.359 | AEE82239 | Phenazine biosynthesis PhzC/PhzF protein [Arabidopsis thaliana] | 2e-45 |
| | LE4F22 | SGN-U579494 | 1.293 | P49201 | 40S ribosomal protein S23-2 | 4e-74 |
| | LE17H22 | SGN-U578426 | 1.228 | AAA34366 | Ribosomal protein L41 | 2e-46 |
| | LE29G13 | SGN-U577703 | 1.175 | P34091 | 60S ribosomal protein L6 | 6e-85 |
| | LE21E17 | SGN-U579293 | 1.067 | O23628 | Histone H2A variant 1 | 6e-53 |
| | LE8P21 | SGN-U584756 | 1.387 | AAR87866 | Ethylene-binding protein [Lycopersicon esculentum] | 9e-136 |
| | LE31D24 | SGN-U564955 | 1.571 | AAO34705 | Ethylene response factor 3 [Lycopersicon esculentum] | 2e-096 |
| | LE29P14 | SGN-U577402 | 1.139 | BAF81889 | Histone H2B [Nicotiana tabacum] | 8e-44 |
| | LE18J20 | SGN-U565168 | 1.114 | BAB20862 | Plastidic cysteine synthase 1 [Solanum tuberosum] | 3e-7 |
| | LE8J06 | SGN-U572337 | 1.870 | BAA21922 | ZPT2-13 [Petunia x hybrida] | 2e-41 |
| | LE6G21 | SGN-U575038 | 1.153 | AEE29872 | PfkB-like carbohydrate kinase family protein [Arabidopsis thaliana] | 1e-123 |
| | LE24O13 | SGN-U580773 | 1.207 | AED96264 | NAD(P)-binding Rossmann-fold superfamily protein [Arabidopsis thaliana] | 9e-109 |
| | LE33K22 | SGN-U563268 | 1.214 | NP_194880 | SDA1 family protein [Arabidopsis thaliana] | 2e-49 |
| | LE12G08 | SGN-U581658 | 1.808 | AAR83861 | Putative lesion-inducing protein [Capsicum annuum] | 1e-57 |
| | LE20E22 | SGN-U580441 | 1.439 | Q9LU64 | Superoxide dismutase [Fe] 2 | 4e-62 |
| | LE14A11 | SGN-U569189 | 1.261 | Q5W274 | Pleiotropic drug resistance protein 3 | 0 |
| | LE25J08 | SGN-U566789 | 1.190 | NP_191425 | 60S ribosomal protein-related [Arabidopsis thaliana] | 8e-81 |
| | LE3E16 | SGN-U575501 | 1.188 | CAH05260 | Steroid 5 alpha reductase DET2 [Solanum lycopersicum] | 2e-153 |
| | LE27O02 | SGN-U575117 | 1.232 | Q9ASY9 | Bifunctional TENA-E protein | 9e-82 |
| | LE2C08 | SGN-U582893 | 1.213 | NP_566986 | Chloroplast lumen common family protein [Arabidopsis thaliana] | 5e-57 |
| | LE12P08 | SGN-U570947 | 1.595 | Q8W2F3 | Transcription factor PIF4 | 2e-29 |
| | LE11P17 | SGN-U566518 | 1.273 | CAO64547 | Unknown protein | 7e-43 |
| | LE9O24 | SGN-U582169 | 1.367 | AAN17752 | Ovate protein [Lycopersicon esculentum] | 1e-138 |
| | LE14P12 | SGN-U582129 | 1.234 | Q9LN71 | E3 ubiquitin-protein ligase | 2e-44 |
| | LE20G16 | SGN-U563415 | 1.293 | Q42948 | Dihydrodipicolinate synthase -related | 4e-173 |
| | LE13H04 | SGN-U595680 | 1.502 | AAT37498 | GDP-mannose pyrophosphorylase identical to GDP-mannose pyrophosphorylase [Arabidopsis thaliana] | 2e-30 |
| | LE4I05 | SGN-U574591 | 1.601 | CAN7784 | Unknown protein | 2e-48 |
| Cluster 4 | | | | | | |
| | LE17G22 | SGN-U566344 | 1.281 | CAN78642 | Unknown protein | 8e-50 |
| | LE12B21 | SGN-U576388 | 1.079 | P19682 | 28 kDa ribonucleoprotein, chloroplastic | 6e-102 |
| | LE25G10 | SGN-U588632 | 1.089 | BAB86842 | Nucleoside diphosphate kinase 3 (ndpk3)[Brassica rapa] | 3e-56 |
| | LE17D11 | SGN-U578905 | 1.359 | Q9SYL9 | 50S ribosomal protein L13, chloroplastic | 6e-93 |
| | LE29L01 | SGN-U581695 | 1.250 | AF118843 | Lycopersicon esculentum ethylene receptor homolog (ETR4) mRNA | 0 |
| | LE5L11 | SGN-U581852 | 1.337 | Q9LW49 | Ethylene-responsive transcription factor 4 | 1e-048 |
| | LE27L22 | SGN-U570991 | 1.206 | Q42463 | Protein DCL, chloroplastic | 6e-103 |
| | LE6I12 | SGN-U571983 | 2.229 | O64882 | Beta-glucosidase 17 | 2e-171 |
| | LE32L04 | SGN-U576004 | 1.248 | Q8L9A0 | 50S ribosomal protein L21, mitochondrial | 2e-47 |
| | LE16D24 | SGN-U585232 | 1.240 | O48670 | Protein RER1A | 6e-71 |
| | LE32L20 | SGN-U577732 | 1.298 | CAH60892 | 1,4-alpha-glucan-maltohydrolase [Lycopersicon esculentum] | 2e-46 |
| | LE29A15 | SGN-U576835 | 1.075 | F4I562 | AP-3 complex subunit mu | 0 |
| | LE3E14 | SGN-U578158 | 1.114 | CAO38748 | Unknown protein | 4e-32 |
| | LE28E23 | SGN-U578376 | 1.132 | AED96512 | RNA recognition motif-containing protein [Arabidopsis thaliana] | 2e-23 |
| | LE22D04 | SGN-U584749 | 1.198 | O82514 | Adenylate kinase | 8e-113 |
| | LE16M02 | SGN-U576146 | 1.227 | CAN82570 | Unknown protein | 8e-163 |
| | LE11N23 | SGN-U578651 | 1.109 | ABA40437 | 40S ribosomal protein S7-like protein [Solanum tuberosum] | 6e-100 |
| | LE30B23 | SGN-U575801 | 1.537 | ABA94758 | hAT family dimerisation domain containing protein [Oryza sativa (japonica cultivar-group)] | 6e-28 |
| | LE21E07 | SGN-U580655 | 1.148 | ABR15768 | Phenylacetaldehyde reductase [Solanum lycopersicum] | 3e-170 |
| | LE31K04 | SGN-U580119 | 2.573 | No hits | No hits | |
| | LE12C04 | SGN-U577073 | 1.661 | CAO63389 | Unknown protein | 3e-55 |
| | LE33K12 | SGN-U570869 | 1.392 | Q9SJY5 | Mitochondrial uncoupling protein 5 | 5e-66 |
| Cluster 5 | | | | | | |
| | LE1F22 | SGN-U580881 | 1.105 | ABB86273 | Cytoplasmic ribosomal protein S13-like [Solanum tuberosum] | 2e-78 |
| | LE16J10 | SGN-U591806 | 1.139 | ABB55398 | 40S ribosomal protein S10-like [Solanum tuberosum] | 1e-30 |
| | LE20K19 | SGN-U572027 | 1.087 | Q9LY66 | 50S ribosomal protein L1 | 5e-115 |
| | LE3L17 | SGN-U579021 | 1.138 | CAN78153 | Unknown protein | 2e-73 |
| | LE32H11 | SGN-U581041 | 1.089 | CAA04703 | Cytochome b5 [Olea europaea] | 1e-62 |
| | LE7D07 | SGN-U564089 | 1.233 | Q9LVY0 | Peptide chain release factor PrfB1 | 7e-165 |
| | LE27G12 | SGN-U564858 | 1.200 | NP_174202 | Ribosomal protein L34 family protein [Arabidopsis thaliana] | 6e-25 |
| | LE8O06 | SGN-U580339 | 1.122 | ABK94898 | Unknown protein | 9e-59 |
| | LE24L22 | SGN-U594635 | 1.174 | No hits | No hits | |
| Cluster 6 | | | | | | |
| | LE21M13 | SGN-U580870 | -0.788 | CAA43590 | Type I (26 kD) CP29 polypeptide [Solanum lycopersicum] | 3e-128 |
| | LE20H03 | SGN-U579535 | -0.824 | AEE31592 | PHD finger protein [Arabidopsis thaliana] | 2e-75 |
| | LE8J10 | SGN-U578475 | -0.720 | Q41480 | Aspartic protease inhibitor 1 | 5e-72 |

| | | | | | | |
|---|---|---|---|---|---|---|
| | LE24H19 | SGN-U573554 | -0.767 | AEE29313 | Lactoylglutathione lyase / glyoxalase I family protein [Arabidopsis thaliana] | 5e-46 |
| | LE10N09 | SGN-U577578 | -0.649 | Q9LW96 | Inositol-3-phosphate synthase | 0 |
| | LE28J17 | SGN-U592121 | -0.737 | Q9STY6 | 40S ribosomal protein S20-2 | 1e-58 |
| | LE31M18 | SGN-U571708 | -0.842 | Q9FPW6 | BTB/POZ domain-containing protein POB1 | 0 |
| | LE6H02 | SGN-U577872 | -0.730 | ABO36637 | Defensin protein [Solanum lycopersicum] | 1e-16 |
| | LE10E22 | SGN-U590165 | -0.836 | AAL49750 | Aquaporin-like protein [Petunia x hybrida] | 2e-93 |
| | LE3I19 | SGN-U573906 | -0.757 | AAM12787 | Putative anthocyanidine rhamnosyl-transferase [Capsicum annuum] | 0 |
| | LE4P11 | SGN-U580025 | -0.914 | A5A7I7 | Calcium-dependent protein kinase 4 | 0 |
| | LE11P21 | SGN-U578553 | -0.857 | ABW74476 | Unknown protein | 9e-43 |
| | LE15K15 | SGN-U585985 | -5.742 | Q9LTS3 | Cytokinin dehydrogenase 3 (CKX3) [Arabidopsis thaliana] | 5e-48 |
| | LE31P22 | SGN-U578375 | -0.678 | No hits | No hits | |
| | LE5K02 | SGN-U579211 | -0.864 | Q944I4 | D-glycerate 3-kinase | 4e-164 |
| | LE28C16 | SGN-U568453 | -0.790 | Q39019 | Shaggy-related protein kinase kappa | 0 |
| | LE30I18 | SGN-U568693 | -0.787 | CAO46087 | Unknown protein | 2e-129 |
| | LE3O13 | SGN-U581813 | -0.775 | AAB40724 | ADP-glucose pyrophosphorylase large subunit [Lycopersicon esculentum] | 0 |
| | LE29P09 | SGN-U578687 | -0.877 | CAO38833 | Unknown protein | 0 |
| | LE5L10 | SGN-U573281 | -0.873 | AAF75794 | 7-transmembrane G-protein-coupled receptor [Solanum chacoense] | 3e-173 |
| | LE25B24 | SGN-U567575 | -0.867 | AF417576_1 | Growth-on protein GRO10 [Euphorbia esula] | 0 |
| | LE13F06 | SGN-U584871 | -0.855 | NP_566244 | Candidate G-protein coupled receptor 2 [Arabidopsis thaliana] | 9e-99 |
| | LE11H12 | SGN-U585309 | -0.804 | O64654 | Thioredoxin-like 1-1 | 1e-60 |
| | LE18G23 | SGN-U565961 | -0.878 | AED90496 | Heavy metal transport/detoxification domain-containing protein [Arabidopsis thaliana] | 3e-33 |
| | LE12N05 | SGN-U570733 | -0.820 | Q9LM15 | Ethylene-responsive transcription factor RAP2-13 | 2e-12 |
| | LE18I06 | SGN-U572920 | -0.683 | CAO61395 | Unknown protein | 2e-96 |
| | LE6O22 | SGN-U573858 | -0.778 | BAE02648 | SEL-1 [Glycine max] | 1e-43 |
| | LE31K19 | SGN-U568494 | -0.789 | Q9LJX4 | Pumilio homolog 5 | 2e-155 |
| | LE2K24 | SGN-U569134 | -0.847 | NP_849999 | Tyrosine decarboxylase 1 [Arabidopsis thaliana] | 0 |
| | LE21M11 | SGN-U567199 | -0.794 | Q9SAK2 | Full=Ent-kaur-16-ene synthase | 4e-73 |
| | LE17A14 | SGN-U571183 | -0.891 | CAO40196 | Unknown protein | 2e-38 |
| | LE1I12 | SGN-U582633 | -0.843 | NP_191845 | Esterase/lipase/thioesterase family protein [Arabidopsis thaliana] | 4e-68 |
| | LE32L05 | SGN-U577050 | -0.740 | No hits | No hits | |
| | LE7C03 | SGN-U582300 | -0.727 | AED91612 | WD and tetratricopeptide repeats protein 1 [Arabidopsis thaliana] | 4e-81 |
| | LE10J10 | SGN-U568120 | -0.896 | O22938 | Leucine-rich repeat receptor-like tyrosine-protein kinase PXC3 | 1e-156 |
| | LE27J13 | SGN-U585803 | -0.873 | ABC87760 | Jasmonic acid-amino acid-conjugating enzyme [Nicotiana attenuata] | 5e-151 |
| | LE28F08 | SGN-U563944 | -0.878 | Q9SKN5 | Auxin response factor 10 | 7e-109 |
| | LE7P03 | SGN-U567636 | -0.786 | No hits | No hits | |
| Cluster 7 | | | | | | |
| | LE14F15 | SGN-U579181 | -0.903 | P07369 | Chlorophyll a/b-binding protein Cab-3C | 1e-147 |
| | LE19J07 | SGN-U583317 | -0.895 | Q9ZT42 | E3 ubiquitin-protein ligase RHF2A | 4e-29 |
| | LE20N01 | SGN-U578302 | -0.843 | ABU40771 | Phytoene synthase 2 [Solanum lycopersicum] | 0 |
| | LE20M17 | SGN-U577253 | -0.904 | P17340 | Plastocyanin | 1e-57 |
| | LE15G20 | SGN-U580357 | -0.924 | CAA70038 | 1,4-alpha-glucan branching enzyme [Solanum tuberosum] | 0 |
| | LE5G07 | SGN-U579166 | -0.930 | AAA34140 | Chlorophyll a/b-binding protein | 4e-118 |
| | LE23F11 | SGN-U579138 | -0.876 | AAO85557 | Photosystem I subunit XI [Nicotiana attenuata] | 1e-93 |
| | LE5A07 | SGN-U579381 | -2.643 | BAA94287 | MADS-box protein pMADS4 [Petunia x hybrida] | 7e-108 |
| | LE8D20 | SGN-U581481 | -5.326 | CAC83066 | MADS-box protein TM29 [Solanum lycopersicum] | 6e-123 |
| | LE12D18 | SGN-U578128 | -0.770 | AAM33098 | TDR4 transcription factor [Lycopersicon esculentum] | 8e-135 |
| | LE29N18 | SGN-U570403 | -0.853 | CAO69028 | Unknown protein | 7e-154 |
| | LE15A19 | SGN-U578526 | -0.601 | Q40168 | Floral homeotic protein AGAMOUS, TAG1 | 5e-138 |
| | LE29D03 | SGN-U564748 | -0.814 | AAY15746 | VIP2 [Nicotiana benthamiana] | 1e-166 |
| | LE13G19 | SGN-U565822 | -0.862 | Q9C9Q8 | Probable pectin methyltransferase QUA2 | 0 |
| | LE32K23 | SGN-U568929 | -2.269 | CAA43171 | TDR6 [Solanum lycopersicum] | 4e-123 |
| | LE20N23 | SGN-U568929 | -2.058 | CAA43171 | TDR6 [Solanum lycopersicum] | 4e-123 |
| | LE26B03 | SGN-U572646 | -9.556 | AAW83046 | CRABS CLAW [Nicotiana tabacum] | 1e-65 |
| | LE27H21 | SGN-U575978 | -0.855 | NP_188563 | Leucine rich repeat protein family [Arabidopsis thaliana] | 2e-99 |
| | LE13N04 | SGN-U573119 | -0.908 | CAO41459 | Unknown protein | 0 |
| | LE3J04 | SGN-U591583 | -0.572 | AAP57412 | MADS-box protein 1 [Lycopersicon esculentum] | 1e-69 |
| | LE19K08 | SGN-U585817 | -0.901 | CAO71339 | Unknown protein | 1e-98 |
| | LE28B23 | SGN-U579099 | -0.757 | P07370 | Chlorophyll a-b binding protein 1B | 3e-144 |
| | LE22H05 | SGN-U570026 | -0.920 | AEE86632 | Serine carboxypeptidase S28 family protein [Arabidopsis thaliana] | 0 |
| | LE12P05 | SGN-U578465 | -0.808 | AEE77006 | Auxin-responsive family protein, AIR12 [Arabidopsis thaliana] | 7e-112 |
| | LE7L15 | SGN-U565270 | -0.813 | CAO40822 | Unknown protein | 4e-27 |
| | LE30L11 | SGN-U574487 | -0.913 | NP_196460 | SWIB complex BAF60b domain-containing protein / plus-3 domain-containing protein / GYF domain-containing protein [Arabidopsis thaliana] | 2e-35 |
| | LE25F14 | SGN-U565180 | -0.611 | Q8L9P8 | RecName: Full=Protein RALF-like 33; Flags: Precursor | 2e-29 |
| | LE2E10 | SGN-U566256 | -0.786 | Q84TH9 | Protein NLP7 | 0 |
| | LE6M22 | SGN-U572674 | -0.747 | Q9LXX5 | PsbP domain-containing protein 6 | 7e-96 |
| | LE13I15 | SGN-U574421 | -0.871 | Q9ZQP1 | Putative dual specificity protein phosphatase DSP8 | 2e-78 |
| | LE9J13 | SGN-U564914 | -0.859 | Q9M3H5 | Probable cadmium/zinc-transporting ATPase HMA1, | 5e-45 |
| | LE4A07 | SGN-U574126 | -0.809 | CAO41465 | Unknown protein | 4e-45 |
| | LE29G02 | SGN-U574613 | -0.804 | BAB09696 | Unknown protein | 4e-70 |
| | LE13C19 | SGN-U565192 | -0.821 | CAO39638 | Unknown protein | 2e-64 |
| | LE1P04 | SGN-U571323 | -0.891 | Q38813 | Chaperone protein dnaJ 1 | 4e-129 |
| | LE14D12 | SGN-U583848 | -0.816 | O80834 | AT-hook motif nuclear-localized protein 9 | 2e-56 |
| | LE27C20 | SGN-U591985 | -2.742 | O22456 | Developmental protein SEPALLATA 3 | 4e-28 |
| Cluster 8 | | | | | | |
| | LE2M11 | SGN-U568770 | -0.547 | P27058 | Systemin | 2e-54 |
| | LE32I09 | SGN-U577258 | -2.966 | AAA80496 | Flower-specific gamma-thionin-like protein [Solanum lycopersicum] | 3e-37 |
| | LE31P14 | SGN-U577283 | -5.681 | NP_200507 | Jasmonic-induced protein 21 (JIP21) [Solanum lycopersicum] | 2e-118 |
| | LE22O19 | SGN-U580463 | -2.148 | P05118 | Wound-induced proteinase inhibitor 1 | 1e-56 |
| | LE12B14 | SGN-U573941 | -0.830 | AEE35435 | Kunitz trypsin inhibitor 1 [Arabidopsis thaliana] | 2e-11 |

| | | | | | |
|---|---|---|---|---|---|
| LE13B19 | SGN-U584909 | -0.505 | AAZ94182 | Proteinase inhibitor I precursor [Solanum tuberosum] | 1e-43 |
| LE26B14 | SGN-U580584 | -0.769 | ABC61504 | AGO4-1 [Nicotiana benthamiana] | 0 |
| LE30P05 | SGN-U568620 | -2.009 | No hits | No hits | |
| LE3C17 | SGN-U565781 | -0.830 | Q9SJM7 | Uridine nucleosidase 1 | 8e-151 |
| LE10B04 | SGN-U576288 | -0.773 | Q9SCV4 | Beta-galactosidase 8 | 3e-178 |
| LE14N18 | SGN-U568745 | -0.869 | AED97350 | RING/U-box domain-containing protein [Arabidopsis thaliana] | 3e-44 |
| LE29B09 | SGN-U569677 | -0.829 | AEE28116 | Neutral/alkaline non-lysosomal ceramidase [Arabidopsis thaliana] | 0 |
| LE23E23 | SGN-U567647 | -0.826 | AAD38941 | 1-D-deoxyxylulose 5-phosphate synthase [Lycopersicon esculentum] | 0 |
| LE17B23 | SGN-U566391 | -0.814 | Q9M331 | Protein NRT1/ PTR FAMILY 5.7 | 1e-94 |
| LE5G17 | SGN-U584480 | -0.894 | CAN81604 | Unknown protein | 3e-91 |
| LE8B14 | SGN-U567779 | -0.747 | BT003007 | Arabidopsis thaliana clone U19136 putative FRO1 and FRO2 protein | 7e-112 |
| LE2P19 | SGN-U578507 | -0.835 | ABF97907 | Unknown protein | 2e-57 |
| LE1F12 | SGN-U569398 | -13.416 | P48007 | Floral homeotic protein PISTILLATA (GLO1) | 4e-52 |
| LE7J23 | SGN-U577905 | -2.835 | AF448521 | Lycopersicon esculentum MADS-box transcription factor MADS-MC (MADS-MC) | 1e-124 |
| LE5H16 | SGN-U566389 | -0.694 | NM_128840 | Arabidopsis thaliana serine/threonine protein kinase AP4.3A mRNA | 2e-103 |
| LE8F20 | SGN-U575492 | -0.576 | NM_104055 | Arabidopsis thaliana IAA-amino acid hydrolase IAR3 mRNA, complete cds | 1e-115 |
| LE18L22 | SGN-U566144 | -0.636 | O65351 | Subtilisin-like protease SBT1.7 | 8e-63 |
| LE17G03 | SGN-U593845 | -0.871 | ABS83388 | Auxin response factor 8 [Solanum lycopersicum] | 1e-26 |
| LE25F06 | SGN-U582930 | -0.877 | P18616 | DNA-directed RNA polymerase II subunit 1 | 0 |
| LE14P09 | SGN-U574087 | -0.857 | Q9ZTX8 | Auxin response factor 6 [Arabidopsis thaliana] | 4e-60 |
| LE10O23 | SGN-U591883 | -0.728 | P07370 | Chlorophyll a-b binding protein 1B, chloroplasti | 1e-82 |
| LE4B10 | SGN-U571612 | -0.833 | Q8L7G0 | Auxin response factor 1 [Arabidopsis thaliana] | 0 |
