# Supplementary material for: A Factor Linking Floral Organ Identity and Growth Revealed by Characterization of the Tomato Mutant unfinished flower development (ufd)
Source: Front Plant Sci. 2016 Nov 7;7:1648. doi: 10.3389/fpls.2016.01648 (PMC5098122; doi:10.3389/fpls.2016.01648)
Supplement: Supplementary file 6 [file Table6.pdf]

Supplementary Table 8 Endogenous hormone concentration (pmol/g) in the flowering shoot apices of *unfinished flower development* (*ufd*) mutant

**Class**	**Compound**	**WT**	***ufd***	
Abscissic acid	Abscisic acid	394.89 ± 70.16	306.65 ± 70.65	ns
	Dihydrophaseic acid	230.57 ± 33.23	277.38 ± 52.41	ns
	Phaseic acid	19.66 ±5.98	51.93 ± 21.68	ns
	ABA-glucose ester	19.16 ± 2.18	13.44 ± 2.09	ns
	Neophaseic acid	7.81 ± 0.97	9.89 ± 2.38	ns
	9-hydroxy-ABA	12.29 ± 4.47	19.27 ± 5.98	ns
Auxins	Indole-3-acetic acid (iaa)	72.71 ± 8.67	128.58 ± 14.94	*
	IAA-aspartate	7.94 ± 0.43	15.10 ± 1.73	*
	IAA-glutamate	1.07 ± 0.10	2.78 ± 0.18	**
	Oxo-IAA	16.79 ± 2.61	24.91 ± 2.85	ns
	Oxo-IAA-glucose ester	8.60 ± 3.64	8.07 ± 0.91	ns
	IAA-glucose ester	72.10 ± 5.23	169.76 ± 45.21	ns
	Phenylacetic acid (PAA)	1092.40 ± 372.59	1645.48 ± 160.69	ns
	Indole-3-acetamide (IAA precursor)	39.60 ± 6.04	57.72 ± 5.67	ns
	Indole-3-acetonitrile (IAA precursor)	0.34 ± 0.14	2.12 ± 0.91	ns
Jasmonates	Jasmonic acid	1941.62 ±3 48.00	2090.40 ± 498.94	ns
	JA-isoleucine	2881.32 ± 449.53	2266.24 ± 245.62	ns
	Cisopda	127.79 ± 14.14	241.53 ± 25.37	*
Gibberellins	Gibberellic acid 8	3.53 ± 1.10	4.81 ± 0.29	ns
	Gibberellic acid 19	5.68 ± 0.36	7.37 ± 0.35	*
Cytokinins	Trans-zeatin	7.04 ± 3.24	13.87 ± 1.00	ns
	Trans-zeatin riboside	87.58 ± 28.83	160.99 ± 43.89	ns
	Trans-zeatin-7-glucoside	67.40 ± 12.43	119.89 ± 8.73	*
	Trans-zeatin-9-glucoside	4.96 ± 1.49	4.23 ± 0.17	ns
	Trans-zeatin-O-glucoside	6.62 ± 3.41	1.37 ± 0.49	ns
	Trans-zeatin riboside -O-glucoside	16.58 ± 12.98	32.10 ± 7.10	ns
	Trans-zeatin riboside monophosphate	3.42 ± 0.34	11.04 ± 0.68	**
	Dihydrozeatin	3.73 ± 1.50	7.28 ± 1.62	ns
	Dihydrozeatin riboside	2.73 ± 1.04	8.34 ± 2.71	ns
	Dihydrozeatin-7-glucoside	76.69 ± 34.27	134.46 ± 19.36	ns
	Dihydrozeatin-9-glucoside	2.43 ± 1.25	0.60 ± 0.15	ns
	Dihydrozeatin riboside -O-glucoside	20.61 ± 7.98	10.63 ± 6.81	ns
	Cis-zeatin	4.04 ± 2.59	6.19 ± 0.27	ns
	Cis-zeatin riboside	49.21 ± 2.67	57.45 ± 4.18	ns
	Cis-zeatin-7-glucoside	7.71 ± 1.29	8.05 ± 1.90	ns
	Cis-zeatin-O-glucoside	0.71 ± 0.34	2.15 ± 1.04	ns
	Cis-zeatin-9-glucoside	1.83 ± 0.55	3.60 ± 0.25	*
	Cis-zeatin riboside -O-glucoside	5.89 ± 3.28	10.44 ± 6.54	ns
	Cis-zeatin riboside monophosphate	1.18 ± 0.19	2.73 ± 0.10	**
	Isopentenyl adenine	0.10 ± 0.10	0.21 ± 0.14	ns
	Isopentenyl adenosine	15.75 ± 3.65	7.84 ± 0.82	ns
	Isopentenyl adenine-7-glucoside	67.32 ± 14.98	73.38 ± 5.44	ns
	Isopentenyl adenine-9-glucoside	1.00 ± 0.16	0.92 ± 0.29	ns
	Isopentenyl adenosine monophosphate	4.48 ± 4.07	1.09 ± 0.12	ns
Ethylene precursor	1-aminocyclopropane-1-carboxylic acid (ACC)	182762 ± 12148	473882 ± 179841	*
Salicylic acid	Salicylic acid	11698 ± 1563	13154 ± 3763	ns
Benzoic acid	Benzoic acid	7002.26 ± 446.87	8318.58 ± 358.43	ns
Strigol	Strigol	635.35 ± 33.49	1351.75 ± 165.66	**

ns, no significant differences, * significant differences at $P < 0.05$, ** significant differences at $P < 0.01$.
