# Supplementary material for: A Factor Linking Floral Organ Identity and Growth Revealed by Characterization of the Tomato Mutant unfinished flower development (ufd)
Source: Front Plant Sci. 2016 Nov 7;7:1648. doi: 10.3389/fpls.2016.01648 (PMC5098122; doi:10.3389/fpls.2016.01648)
Supplement: Supplementary file 7 [file Image1.PDF]

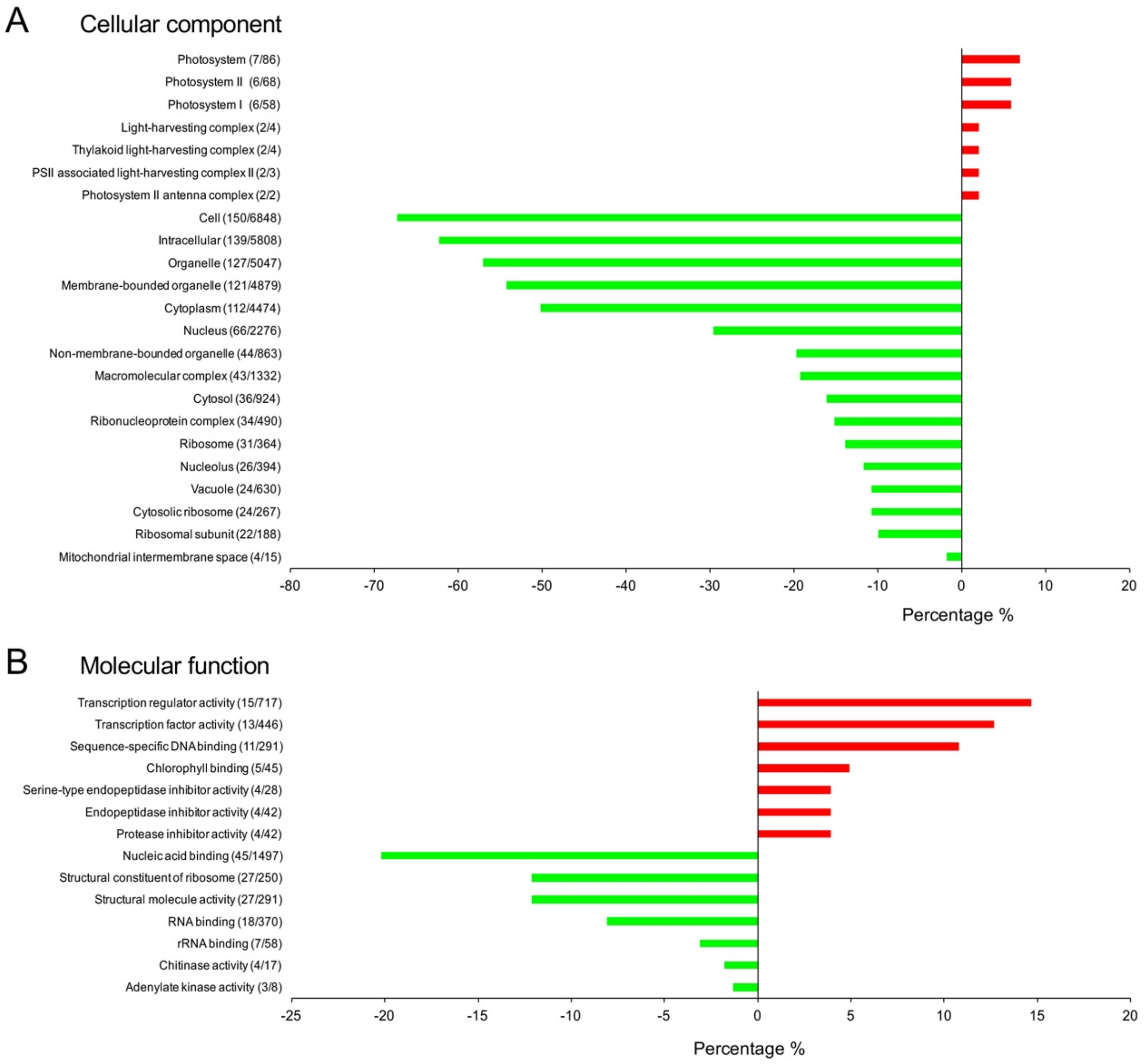

Supplementary Fig. 1 The impact of the *unfinished flower development (ufd)* mutation on different categories of genes based on Gene Ontology (GO) term analysis using Tomato Functional Genomics Database (TFGD, http://ted.bti.cornell.edu/) tools. The percentages of differentially expressed genes in *ufd*, classified either by cellular component (**A**) and molecular function (**B**). Red or green bar indicates up- or down-regulated categories, respectively. In parenthesis is shown the number of deregulated genes in each category respect to the total number of genes within that category in the tomato genome (for more information see Supplementary Tables 3 and 4).
